# A Calcipotriol-Responsive ZF10–ΔVDR Switch for Mammalian Transgene Regulation with Topical Induction In Vivo

**DOI:** 10.3390/genes17091072

**Published:** 2026-09-04

**Authors:** Hong Su, Shiting Li, Guanyang Chen, Peng Bai

**Affiliations:** 1 Department of Laboratory Medicine, The First Affiliated Hospital, Sun Yat-sen University, Guangzhou 510080, China; suh2408@outlook.com (H.S.);; 2 Institute of Precision Medicine, The First Affiliated Hospital, Sun Yat-sen University, Guangzhou 510080, China; 3 Greater Bay Area Institute of Precision Medicine (Guangzhou), School of Life Sciences, Fudan University, Shanghai 200438, China

**Keywords:** synthetic gene switch, pharmacological control, mammalian synthetic biology, transgene regulation, gene and cell therapy

## Abstract

Background: Small-molecule-regulated gene switches are important tools for programmable mammalian cell engineering, and compact switches responsive to clinically familiar ligands that can be administered locally are particularly attractive for externally regulated transgene expression. Methods: Here, we developed ZF10–ΔVDR, a compact zinc-finger–VDR transcriptional switch, by fusing an orthogonal human-derived zinc-finger array, ZF10, to the hinge and ligand-binding domain of the vitamin D receptor. The resulting switch enabled transgene regulation by calcipotriol, a clinically used topical vitamin D analog and VDR agonist. Results: ZF10–ΔVDR maintained low basal activity, showed limited activation by the tested vitamin D species at physiologically relevant reference concentrations, and was robustly activated by calcipotriol. The system functioned across multiple human cell types. In vivo, topical calcipotriol increased serum reporter output in mice bearing subcutaneous microencapsulated-cell implants, whereas oral vitamin D3 did not measurably activate this system under the tested conditions. In a separate HaCaT implantation model, topical calcipotriol increased bioluminescence at wound-adjacent implantation sites. ZF10–ΔVDR also supported reversible ON/OFF cycling under the tested conditions and inducible secreted protein output in vitro. Conclusions: These findings establish ZF10–ΔVDR as a calcipotriol-responsive switch with limited activation by endogenous vitamin D species, activity across multiple mammalian cell types, reversible regulation under the tested conditions, and compatibility with topical induction in vivo.

## 1. Introduction

Synthetic biology provides powerful strategies to engineer mammalian cells with programmable functions [1,2,3,4,5,6]. Central to this approach are gene-regulatory switches that couple small-molecule cues to conditional transgene expression. Existing switches have been developed using diverse transcription-factor-, receptor-signaling-, RNA-, and promoter-based architectures, each with distinct advantages for different applications [7,8,9,10,11,12,13]. Nevertheless, additional compact gene control modules that combine mammalian compatibility, low basal activity, and responsiveness to clinically familiar ligands would broaden the toolkit for externally regulated mammalian transgene expression.

Recent studies have advanced human-compatible gene control strategies at the protein, RNA, and promoter levels [7,8,9,10,11,12,13,14]. These efforts highlight the value of modular switches that integrate suitable ligand properties with delivery-compatible genetic components. Because several widely used transcription-factor-based systems, including Tet-On/Tet-Off and Gal4-UAS variants, rely on non-human components such as bacterial TetR-derived regulators, yeast GAL4 DNA-binding domains, and viral VP16-derived activation domains in commonly used transactivators [15,16,17], transcriptional designs built from human-derived domains are attractive for reducing potential immunogenicity concerns in engineered-cell applications [7,8,18,19].

Zinc finger proteins (ZFPs) are the most abundant family of transcription factors in the human genome and provide compact, programmable DNA-binding modules [20,21,22]. Each approximately 30 amino acid domain can be assembled into arrays with tunable specificity, enabling applications in genome editing and synthetic transcriptional control [20,21,22]. Importantly, zinc finger arrays can be designed to recognize synthetic DNA sequences with minimal predicted overlap with the human genome, thereby reducing potential crosstalk with host transcription. The synZiFTR framework demonstrated the feasibility of orthogonal zinc finger-based transcriptional regulators and highlighted their potential for human-compatible gene control [7]. Earlier work also established chemically regulated zinc-finger transcription factors and humanized pharmacologic gene control systems [19,23].

Nuclear receptor (NR) ligand-binding domains provide a rich source of compact, small-molecule-responsive sensing modules [24,25]. However, a major challenge for NR-based switches is interference from endogenous ligands or metabolites. Their ligand-binding domains may respond to physiological molecules at low-nanomolar concentrations, resulting in baseline activity that can complicate precise control [24,25]. For the vitamin D receptor (VDR), this issue is particularly relevant because multiple endogenous vitamin D species circulate in vivo. We therefore sought to engineer a VDR-based switch that separates ligand sensing from native VDR DNA recognition, remains minimally responsive to physiological vitamin D metabolite levels, and can be activated by a clinically used ligand suitable for topical administration.

Calcipotriol is a synthetic vitamin D_3_ analog and VDR agonist widely used topically for dermatologic therapy [26,27]. Its established topical use makes it attractive as a non-invasive inducer for accessible implantation sites or engineered-cell applications requiring external control. ZF10 was adopted from the previously characterized synZiFTR platform. Its cognate synthetic DNA-binding sequence was selected to minimize predicted matches in the human genome, and representative ZF10-based regulators showed limited perturbation of the native human transcriptome [7]. Here, we developed a compact calcipotriol-responsive transcriptional regulator with an orthogonal ZF10–P_ZF10_ promoter-targeting module by fusing a human-derived ZF10 DNA-binding array [7] to the hinge and ligand-binding domain of VDR. This design removes the native VDR DNA-binding domain to avoid direct recognition of VDR response elements and deletes the N-terminal activation domain to reduce ligand-independent activation. The resulting ZF10–ΔVDR switch uses ZF10 to bind a synthetic promoter, P_ZF10_, containing ZF10-binding motifs linked to a minimal promoter, thereby coupling calcipotriol exposure to transgene expression.

In this study, we show that ZF10–ΔVDR maintains low basal activity, responds to low-nanomolar calcipotriol, and shows limited activation at physiologically relevant concentrations of the tested endogenous vitamin D species. We validate this switch across multiple human and murine cell types, including primary human adipose-derived stromal cells and skin-associated HaCaT keratinocytes, and demonstrate reversible ON/OFF cycling in vitro. In vivo, topical calcipotriol increased reporter output in mice bearing subcutaneous microencapsulated-cell implants, whereas oral vitamin D3 did not measurably activate the system under the tested conditions. In a separate model using HaCaT cells implanted adjacent to cutaneous wounds, topical calcipotriol increased bioluminescence at the implantation site. These findings establish ZF10–ΔVDR as a compact calcipotriol-responsive transcriptional switch for mammalian transgene regulation and demonstrate its compatibility with topical induction in implanted-cell settings.

## 2. Materials and Methods

### 2.1. Plasmid Construction

All plasmids used in this study are listed in Supplementary Table S1. The plasmids used in each figure are summarized in Supplementary Table S4. Plasmids were constructed using either seamless cloning (homology-directed) or restriction enzyme-based digestion and ligation. All procedures were carried out according to the manufacturers’ instructions. ZF10–ΔVDR comprises the six-finger ZF10 DNA-binding array fused to the human VDR fragment spanning residues 90–427, encompassing the hinge and ligand-binding regions. P_ZF10_ contains eight tandem ZF10-binding motifs arranged in the same orientation upstream of the minimal ybTATA promoter. The ZF10–ΔVDR coding sequence is 1548 bp in length, including the stop codon, and encodes a 515-amino-acid fusion protein. Complete DNA sequences of key constructs are provided in Supplementary Table S2.

### 2.2. Reagents

Calcipotriol (MCE, Monmouth Junction, NJ, USA, HY-10001-10 mg), calcitriol (MCE, HY-10002-2 mg), calcifediol (MCE, HY-32351-1 mg), vitamin D3 (MCE, HY-15398-100 mg) and grazoprevir (GZV; MCE, HY-15298-1 mg) were prepared in DMSO (MP Biomedicals, Irvine, CA, USA, 0219605580) for in vitro experiments. Stock solutions were protected from light where appropriate and stored at −80 °C or −20 °C until use. Calcipotriol and calcifediol stocks were aliquoted before storage, whereas calcitriol and vitamin D3 working solutions were prepared freshly before use. GZV was prepared in DMSO and stored frozen until use. Recombinant human hepatocyte growth factor (HGF) (NovoProtein, Suzhou, China, CJ72-10 ug) was reconstituted according to the manufacturer’s instructions, aliquoted, and stored at −80 °C until use.

For in vivo studies, calcitriol and vitamin D3 were dissolved directly in corn oil (Macklin Biochemical Co., Ltd., Shanghai, China; No. C805618) for intraperitoneal injection or oral gavage, respectively. Calcipotriol was administered topically in two formulations. An ethanol-based solution was prepared by diluting a 10 mM DMSO stock with ethanol and was applied dropwise to the shaved dorsal skin. A commercial calcipotriol ointment (Daivonex, LEO Laboratories Limited, Dublin, Ireland; approval HJ20160070) was also applied directly at defined doses.

### 2.3. Cell Culture

HEK293T (RRID: CVCL_0063; Cell Bank of Chinese Academy of Sciences, National Collection of Authenticated Cell Cultures [NCACC], Shanghai, China; GNHu17), human keratinocyte cell line HaCaT (RRID: CVCL_0038; NCACC; GNHu64), and hTERT A41hBAT-SVF (RRID: CVCL_UI86; ATCC, Manassas, VA, USA, CRL-3385) were cultured in Dulbecco’s modified Eagle’s medium (DMEM, HyClone, Logan, UT, USA, SH30243.01) supplemented with 10% (*v*/*v*) fetal bovine serum (FBS, VisTech, Sydney, NSW, Australia, SE100-B) and 1% (*v*/*v*) penicillin–streptomycin solution (P/S, Biosharp, Beijing, China, BL505A).

MIN6 cells (RRID: CVCL_0431; BNCC, Beijing, China, BNCC352010) were cultured in DMEM supplemented with 15% (*v*/*v*) embryonic stem cell-qualified FBS (VisTech, SE200-ES), 1% (*v*/*v*) P/S, 20 mM HEPES (Meilunbio, Dalian, China, MA0036), and 55 μM 2-mercaptoethanol (Sigma-Aldrich, St. Louis, MO, USA, M3148-25 mL). HMEC-1 cells (RRID: CVCL_0307; NCACC; GNHu46) were cultured in MCDB 131 medium (Gibco, Grand Island, NY, USA, 10372019) supplemented with 10% (*v*/*v*) FBS, 1% (*v*/*v*) P/S, and 10 ng/mL human EGF (Sino Biological, Beijing, China, 10605-HNAE-100).

Primary human adipose-derived stromal cells (ADSCs) (OriCell, Guangzhou, China, HUXMD-01001) were cultured in DMEM/F-12 (Gibco, C11330500BT) supplemented with 10% (*v*/*v*) ES-qualified FBS (VisTech), 1% (*v*/*v*) P/S (Biosharp), 5 ng/mL hEGF (Sino Biological), and 5 ng/mL hFGF2 (Sino Biological, 10014-HNAE-10). All cells were maintained at 37 °C in a humidified incubator with 5% CO_2_ and were routinely tested for mycoplasma contamination, with negative results.

### 2.4. RT-qPCR Analysis

Total RNA was isolated from the indicated cells and reverse-transcribed into cDNA. Relative expression of *CYP24A1* and *CAMP* was quantified by RT-qPCR using *GAPDH* as the reference gene. Primer sequences are provided in Supplementary Table S3.

### 2.5. Transient Transfection

HEK293T cells were transfected using polyethyleneimine (PEI; Polysciences, Warrington, PA, USA, 24765-1) according to an optimized protocol. Briefly, cells were seeded in 6 cm dishes and cultured overnight to 70–80% confluence. Cells were subsequently co-transfected with the corresponding plasmid mixtures at a 1:1 DNA mass ratio, using a total of 4 µg DNA per 6 cm dish, for 8–12 h using PEI (PEI:DNA mass ratio = 3:1); a total of 200 µL PEI–DNA mixture was added per 6 cm dish. Cells were treated with the indicated compounds after medium replacement, and supernatants were collected for reporter assays.

### 2.6. Lentivirus Production and Generation of Stable Cell Lines

Lentiviral particles were generated in HEK293T cells by co-transfection with psPAX2, pMD2.G, and the transfer plasmid. Cells were seeded in 10 cm dishes and transfected at 80–90% confluence for 8–12 h using PEI (Polysciences) at a PEI:DNA mass ratio of 3:1. For each 10 cm dish, 1.3 pmol psPAX2, 0.72 pmol pMD2.G, and 1.64 pmol transfer plasmid were used, with a total transfection mixture volume of 0.5 mL per dish. Viral supernatants were collected 48–72 h post-transfection, centrifuged at 1000× *g* for 10 min to remove cell debris, and filtered through a 0.45 µm PVDF filter (MilliporeSigma, Burlington, MA, USA; No. SLHVR33RB). Aliquots were stored at 4 °C for short-term use or at −80 °C for long-term storage.

For adherent cell lines, cells were seeded in 6 cm dishes at 50–60% confluence and infected with 5 mL viral supernatant supplemented with 8 µg/mL polybrene (Sigma-Aldrich, St. Louis, MO, USA; No. TR-1003-G). After 4 h, an equal volume of complete medium was added and cells were subcultured 24–48 h post-infection. Stable populations were selected with puromycin (0.2–2 µg/mL; InvivoGen, San Diego, CA, USA, ant-pr-1) or blasticidin (2–20 µg/mL; InvivoGen, ant-bl-1) for 7 days, depending on the construct and cell type.

### 2.7. SEAP Assay

SEAP activity in culture supernatants was quantified by kinetic measurement of p-nitrophenyl phosphate (pNPP) hydrolysis. Supernatants were heat-inactivated at 65 °C for 30 min, and 80 µL of each was mixed with 100 µL of 2× assay buffer (1 mM MgCl_2_, 2 M diethanolamine, 2 mM L-homoarginine, pH 9.8) and 20 µL p-nitrophenyl phosphate (120 mM; Aladdin, Shanghai, China, P109039-100 g). Absorbance at 405 nm was recorded kinetically at 37 °C on a Varioskan LUX microplate reader (Thermo Fisher Scientific, Vantaa, Finland). SEAP activity was calculated from the slope (ΔA405/min) of the linear portion of the kinetic trace using a previously described pNPP-based SEAP assay [28,29].

Serum SEAP was quantified using the SEAP Reporter Gene Assay Kit (Abcam, Cambridge, UK, ab133077) following the manufacturer’s instructions. Samples (20 µL) were heat-inactivated at 65 °C for 30 min, and 10 µL was mixed with 50 µL of kit substrate in white 96-well plates. Chemiluminescence was recorded on a Varioskan LUX microplate reader under identical measurement settings and expressed as relative luminescence units (RLU).

### 2.8. Luciferase Assay

**Figure 1:**
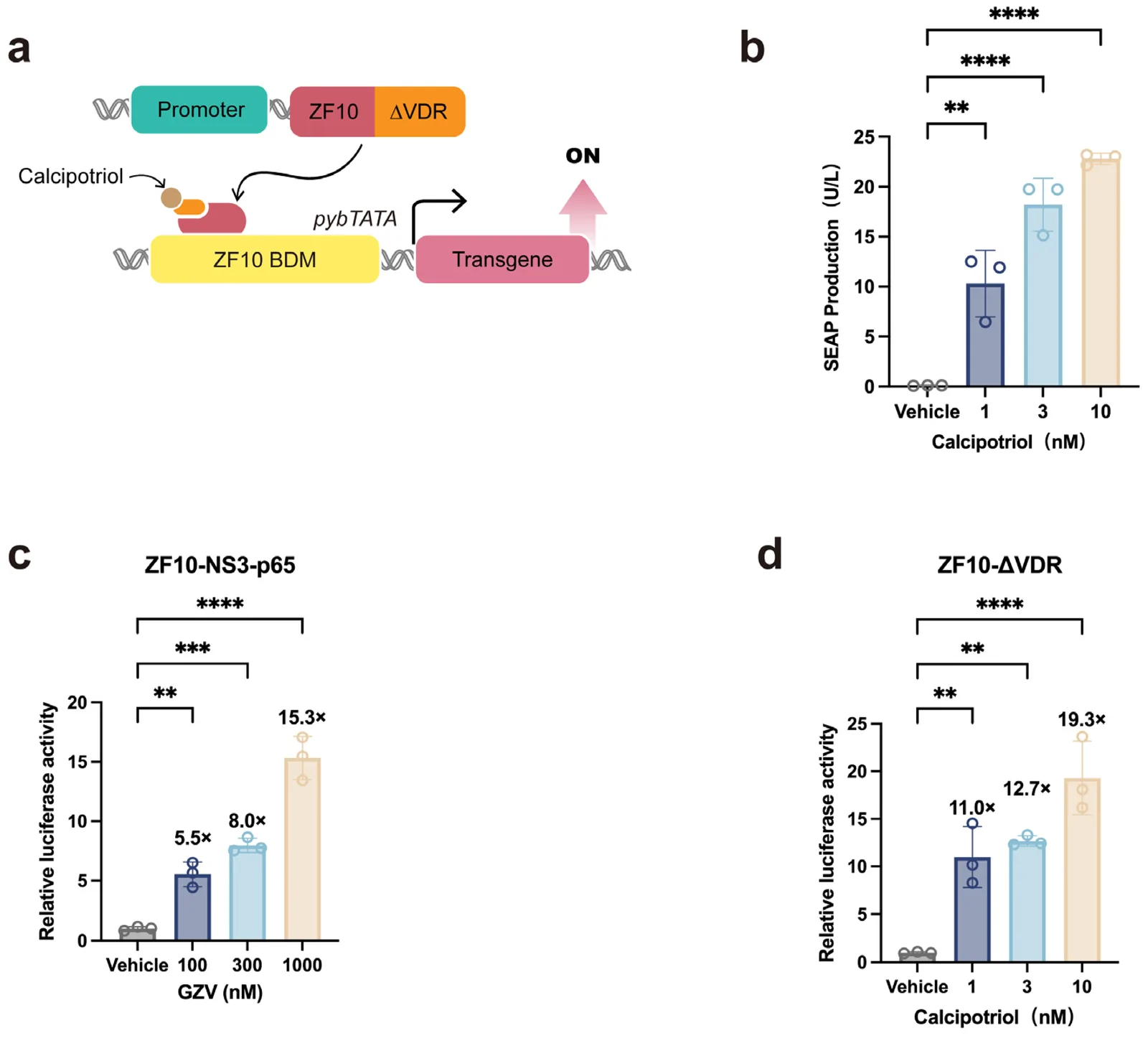
Construction and initial characterization of the calcipotriol-inducible ZF10–ΔVDR switch. (**a**) Conceptual schematic of the ZF10–ΔVDR system. A truncated VDR fragment comprising the hinge and ligand-binding domain was fused to the ZF10 zinc-finger array to generate a calcipotriol-responsive transactivator. ZF10 serves as the promoter recognition module for the synthetic P_ZF10_ promoter, which contains eight tandem ZF10-binding motifs upstream of a minimal ybTATA promoter, whereas the retained VDR-derived region confers calcipotriol responsiveness. The cognate ZF10–ΔVDR/P_ZF10_ configuration supports ligand-dependent transgene expression. The schematic illustrates the modular architecture of the switch and does not imply that calcipotriol alters promoter occupancy. (**b**) Dose–response of ZF10–ΔVDR to calcipotriol. HEK293T cells were co-transfected with P_ZF10_–SEAP and ZF10–ΔVDR, treated with increasing concentrations of calcipotriol, and SEAP activity was quantified after 24 h. Data are presented as mean ± SD (*n* = 3 biologically independent samples). (**c**,**d**) Functional comparison of two ligand-responsive transactivators operating through the ZF10/P_ZF10_ promoter recognition framework. HEK293T cells expressing the indicated transactivator were evaluated using the P_ZF10_–firefly luciferase reporter and a Renilla luciferase normalization control. (**c**) ZF10–NS3–p65 cells were treated with vehicle or grazoprevir (GZV; 100, 300, or 1000 nM). (**d**) ZF10–ΔVDR cells were treated with vehicle or calcipotriol (1, 3, or 10 nM). Firefly luciferase activity was measured after 24 h. Firefly luciferase activity was normalized to Renilla luciferase activity and expressed relative to the corresponding vehicle condition. Fold induction is indicated above the bars. Data are presented as mean ± SD from three independent experiments. Statistical significance was determined by one-way ANOVA followed by Dunnett’s multiple comparisons test; ** *p* < 0.01, *** *p* < 0.001, **** *p* < 0.0001.

Adherent cells were washed twice with PBS and lysed by adding 20 µL lysis buffer per well. Plates were shaken vigorously for 10–15 min until complete lysis. Then, 5–10 µL of lysate were transferred to a white 96-well plate. Luciferase reagent (50 µL) was added immediately and chemiluminescence was read on a Varioskan LUX (Thermo Fisher Scientific). Background from blank controls was subtracted. Procedures followed the Dual Luciferase Reporter Assay Kit (Vazyme, Nanjing, China, DL101-01). For the comparison experiments shown in Figure 1c,d, Renilla luciferase was co-measured and firefly luciferase activity was normalized to the corresponding Renilla signal. In contrast, for experiments evaluating ZF10–ΔVDR function across multiple mammalian cell types (Figure 3a–c, Supplementary Figures S1c,g,k and S2a,b), only firefly luciferase activity was measured.

### 2.9. CCK-8 Assay

CCK-8 assays were performed using a Cell Counting Kit-8 (CCK-8; ECOTOP SCIENTIFIC, Guangzhou, China; Cat. No. EK-5103) according to the manufacturer’s instructions. Absorbance was measured at 450 nm using a microplate reader. CCK-8 signals were expressed relative to the corresponding vehicle-treated condition and used as an indicator of viable-cell-associated metabolic activity.

### 2.10. HGF ELISA and Transwell Cell Migration Assay

Human HGF concentrations in culture supernatants were measured using a human HGF ELISA kit (Neobioscience, Shenzhen, China, EHC138.96) according to the manufacturer’s instructions.

Cell migration was assessed using Transwell chambers (8 µm, Beyotime, Shanghai, China, FTW076-48Ins). Briefly, HEK293T cells were seeded in the lower chamber at 5 × 10^4^ cells per well, and HaCaT cells were seeded in the upper chamber at 1 × 10^5^ cells per insert. Where indicated, recombinant human HGF was added to the lower chamber at the indicated concentration as a positive control. After co-culture, non-migrated cells on the upper surface of the membrane were removed, and cells that had migrated to the lower surface were fixed with 4% paraformaldehyde (Guangzhou Zhuanyan Biotechnology Co., Ltd., Guangzhou, China; No. SRB002), stained with 0.1% crystal violet (Beyotime, No. C0121), and observed under a microscope.

### 2.11. Animal Experiments

C57BL/6J mice and BALB/c Nude mice were purchased from Guangdong GemPharmatech (Foshan, China) and housed in a specific pathogen-free facility. Male C57BL/6J mice aged 6–8 weeks were used for the microencapsulation studies, whereas female BALB/c nude mice aged 8–10 weeks were used for the wound-associated HaCaT implantation study. All animal experimental procedures were approved by the Institutional Animal Care and Use Committee of Sun Yat-sen University (Approval Code: SYSU-IACUC-2024-002668 (Application No. 2024002499); SYSU-IACUC-2025-001527 (Application No. 2025001555)). Animals were monitored after implantation and wound procedures for general condition, wound status, and signs of distress. Humane endpoints followed the criteria specified in the approved institutional animal protocols.

Engineered HEK293T cells were encapsulated in alginate–poly-L-lysine–alginate (APA) microbeads using an Encapsulator B-395 Pro (BUCHI, Flawil, Switzerland) with a 200 µm nozzle (1025 Hz vibration; 1.12 kV dispersion voltage).

To evaluate transgene activation in vivo, engineered cells were encapsulated in APA microbeads before administration. A total of 2 × 10^6^ cells per mouse was delivered by intraperitoneal or dorsal subcutaneous injection. For male C57BL/6J mice receiving microcapsules, injection volumes were 500 µL for intraperitoneal injection and 300 µL for subcutaneous injection.

Following intraperitoneal injection of cells, mice were treated once daily for two consecutive days with calcitriol (0.1 µg per mouse, intraperitoneally) in corn oil. After subcutaneous administration of cells, the dorsal skin received topical treatment with calcipotriol in ethanol-based formulations at 0.6 or 4 nmol per application, twice daily for two consecutive days. DMSO was matched across the corresponding treatment and vehicle groups. Commercial calcipotriol ointment (0.005% *w*/*w*; 37 mg per application) was also applied twice daily for two days. A formulation-matched ointment base was not available for the commercial ointment group. Vitamin D3 (25 µg/kg, oral gavage) was administered once daily for two days where indicated. Blood was collected by orbital bleeding at 24 h or 48 h post-cell injection for serum SEAP measurement.

### 2.12. Wound-Associated HaCaT Implantation and In Vivo Bioluminescence Imaging

HaCaT keratinocytes stably expressing ZF10–ΔVDR and carrying the P_ZF10_–firefly luciferase reporter were harvested and counted. For each mouse, 2 × 10^6^ cells were resuspended in 50 µL of a 1:1 mixture of PBS and Matrigel (Corning Incorporated, Corning, NY, USA; No. 354234) and maintained on ice until implantation. BALB/c-nude mice were anesthetized, and the dorsal skin was disinfected with ethanol. A 6 mm circular cutaneous wound was generated using a biopsy punch without disrupting the underlying fascia. A circular splint with an inner diameter of 6 mm and an outer diameter of 8 mm was positioned around the wound, fixed with tissue adhesive, and covered with a transparent dressing. The cell suspension was injected subcutaneously adjacent to the wound margin and allowed to solidify for several minutes.

Calcipotriol was applied topically to the wound region at 4 nmol in 20 µL per application, twice daily for two consecutive days. Control mice received the corresponding vehicle according to the same schedule. On day 2 after cell implantation, mice received D-luciferin sodium salt (Yeasen Biotechnology, Shanghai, China; No 40901ES) intraperitoneally at 150 mg/kg. Mice were anesthetized with isoflurane (RWD Life Science, Shenzhen, China; No. R510-22-10), and bioluminescence images were acquired using a PerkinElmer IVIS Spectrum imaging system with a 30 s exposure and binning of 8. A region of interest encompassing the wound and adjacent implantation site was used for all animals, and bioluminescence was quantified as total flux (photons/s). Image datasets were anonymized, and ROI placement and quantification were performed by an investigator blinded to treatment allocation.

### 2.13. Statistical Analysis

**Figure 2:**
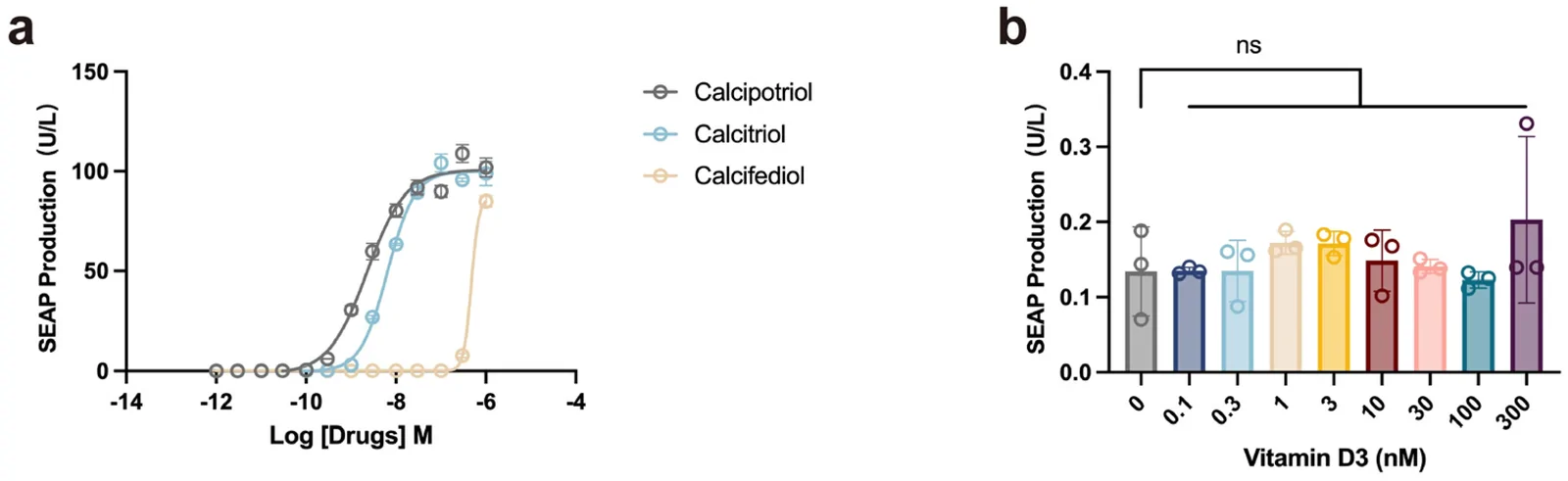
Profiling of ZF10–ΔVDR responses to calcipotriol and endogenous vitamin D species. (**a**) Matched concentration–response profiles of ZF10–ΔVDR to calcipotriol, calcitriol, and calcifediol in HEK293T cells stably carrying P_ZF10_–SEAP and ZF10–ΔVDR. Cells were treated for 24 h with increasing concentrations of the indicated ligands, and SEAP activity was quantified. The fitted EC50 values were 2.28 nM for calcipotriol and 6.51 nM for calcitriol; Fitted parameters are provided in Supplementary Table S5. Data are presented as mean ± SD (*n* = 3 independent experiments). (**b**) SEAP production in the same stable HEK293T cells after stimulation for 24 h with increasing concentrations of vitamin D3 (cholecalciferol). Data are presented as mean ± SD (*n* = 3 independent experiments). Statistical significance was determined by one-way ANOVA followed by Dunnett’s multiple comparisons test; ns, not significant.

**Figure 3:**
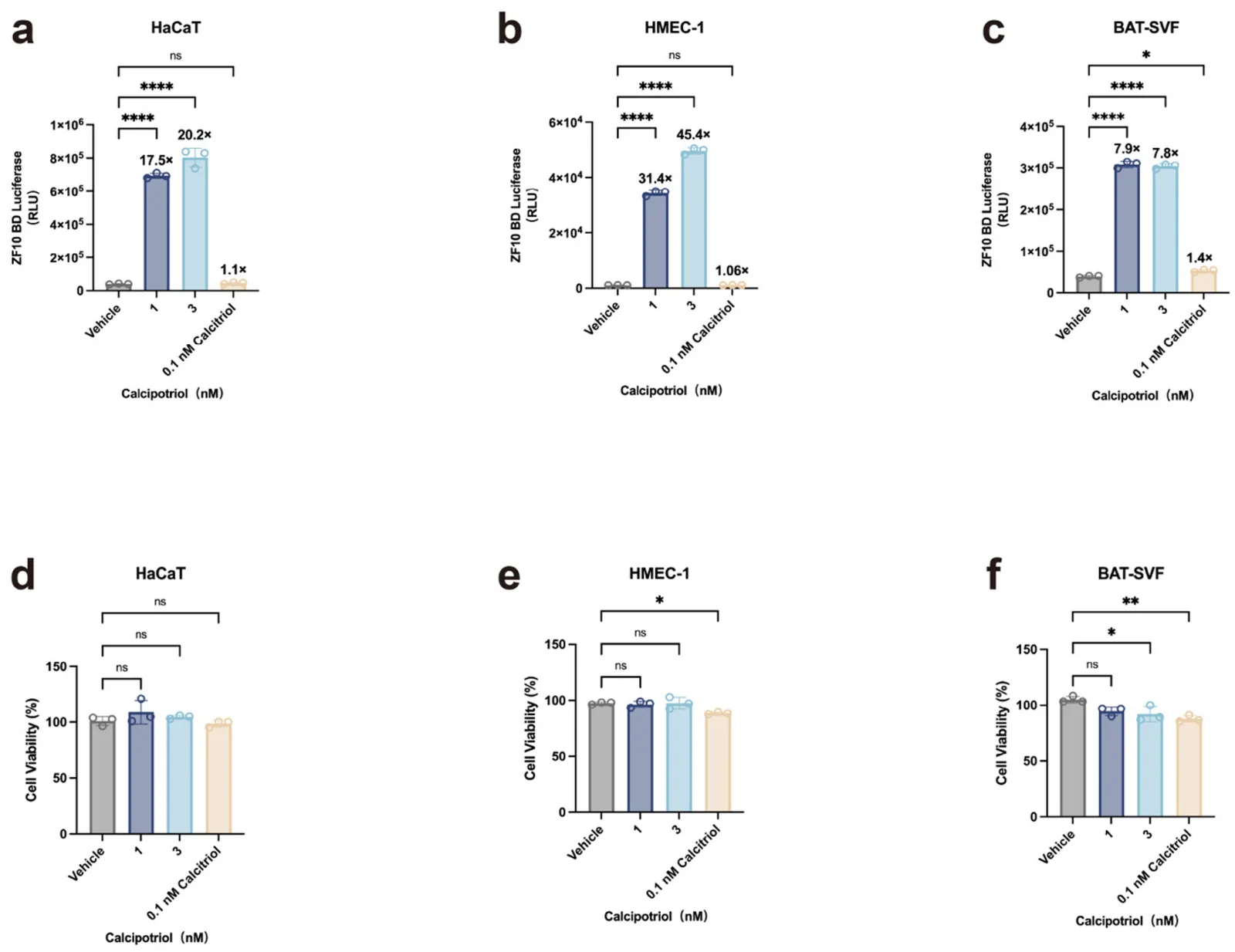
Functional validation of ZF10–ΔVDR in representative human cell types. Stable HaCaT (**a**,**d**), HMEC-1 (**b**,**e**), and hTERT A41h-BAT-SVF (**c**,**f**) populations carrying ZF10–ΔVDR and the P_ZF10_–luciferase reporter were treated with calcipotriol (1 or 3 nM), 0.1 nM calcitriol, or vehicle for 24 h. (**a**–**c**) Firefly luciferase activity. (**d**–**f**) CCK-8 signals measured in matched parallel wells and expressed relative to the corresponding vehicle condition. Data are presented as mean ± SD (*n* = 3 independent experiments). Statistical significance was determined by one-way ANOVA followed by Dunnett’s multiple-comparisons test against vehicle; ns, not significant; * *p* < 0.05; ** *p* < 0.01; **** *p* < 0.0001.

**Figure 4:**
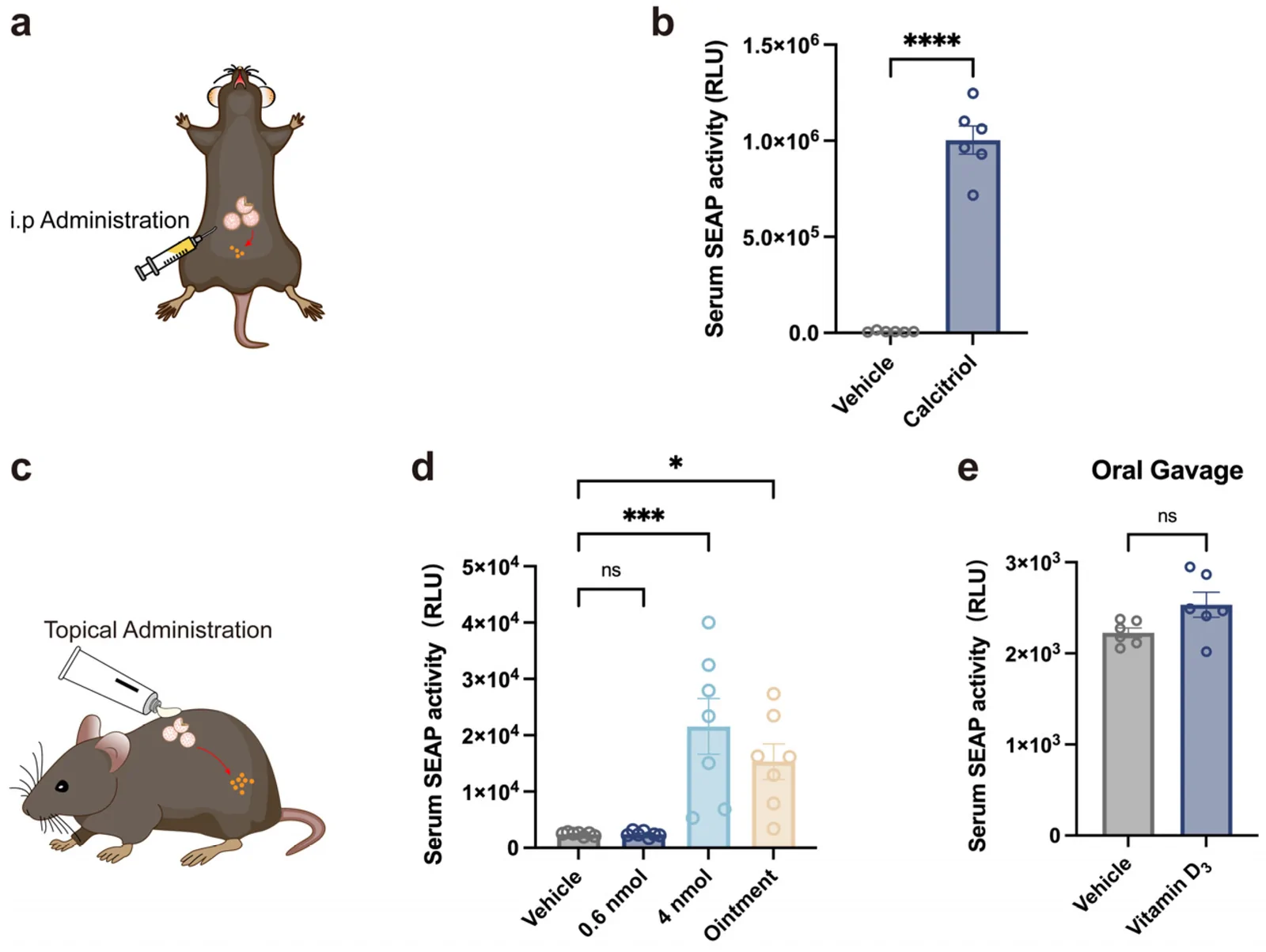
Systemic and topical regulation of ZF10–ΔVDR in vivo. (**a**) Schematic of calcitriol-mediated activation of ZF10–ΔVDR in male C57BL/6J mice implanted intraperitoneally with microencapsulated reporter cells. (**b**) Microencapsulated HEK293T cells stably carrying the P_ZF10_–SEAP reporter and transiently transfected with ZF10–ΔVDR were transplanted intraperitoneally into mice (2 × 10^6^ cells per mouse). Mice received calcitriol (0.1 μg per mouse, i.p., once daily for two consecutive days). Serum SEAP activity was measured 48 h after treatment initiation by chemiluminescence and expressed as relative luminescence units (RLU). Data are presented as mean ± SEM (*n* = 6 mice; each point represents one mouse). Statistical significance was determined using an unpaired two-sided *t* test. **** *p* < 0.0001. (**c**) Schematic of topical calcipotriol-mediated activation of ZF10–ΔVDR in male C57BL/6J mice implanted subcutaneously with microencapsulated reporter cells. (**d**,**e**) Microencapsulated HEK293T cells stably carrying the P_ZF10_–SEAP reporter and transiently transfected with ZF10–ΔVDR were transplanted subcutaneously into mice (2 × 10^6^ cells per mouse). (**d**) Mice received topical application of calcipotriol ointment (37 mg per application, twice daily) or an ethanol-based calcipotriol solution (0.6 or 4 nmol per application, twice daily). A formulation-matched ointment-base control was not available for the commercial calcipotriol ointment group. * *p* < 0.05; *** *p* < 0.001. (**e**) Mice received vitamin D3 by oral gavage (25 μg/kg, once daily) or the corresponding vehicle. Treatments were administered for two consecutive days. Serum SEAP activity was measured 48 h after treatment initiation by chemiluminescence and expressed as RLU. Data are presented as mean ± SEM (*n* = 6–7 mice per group; each point represents one mouse). Statistical significance was determined by one-way ANOVA followed by Dunnett’s multiple-comparisons test against vehicle for panel (**d**) and an unpaired two-sided *t* test for panel (**b**,**e**). ns, not significant.

**Figure 5:**
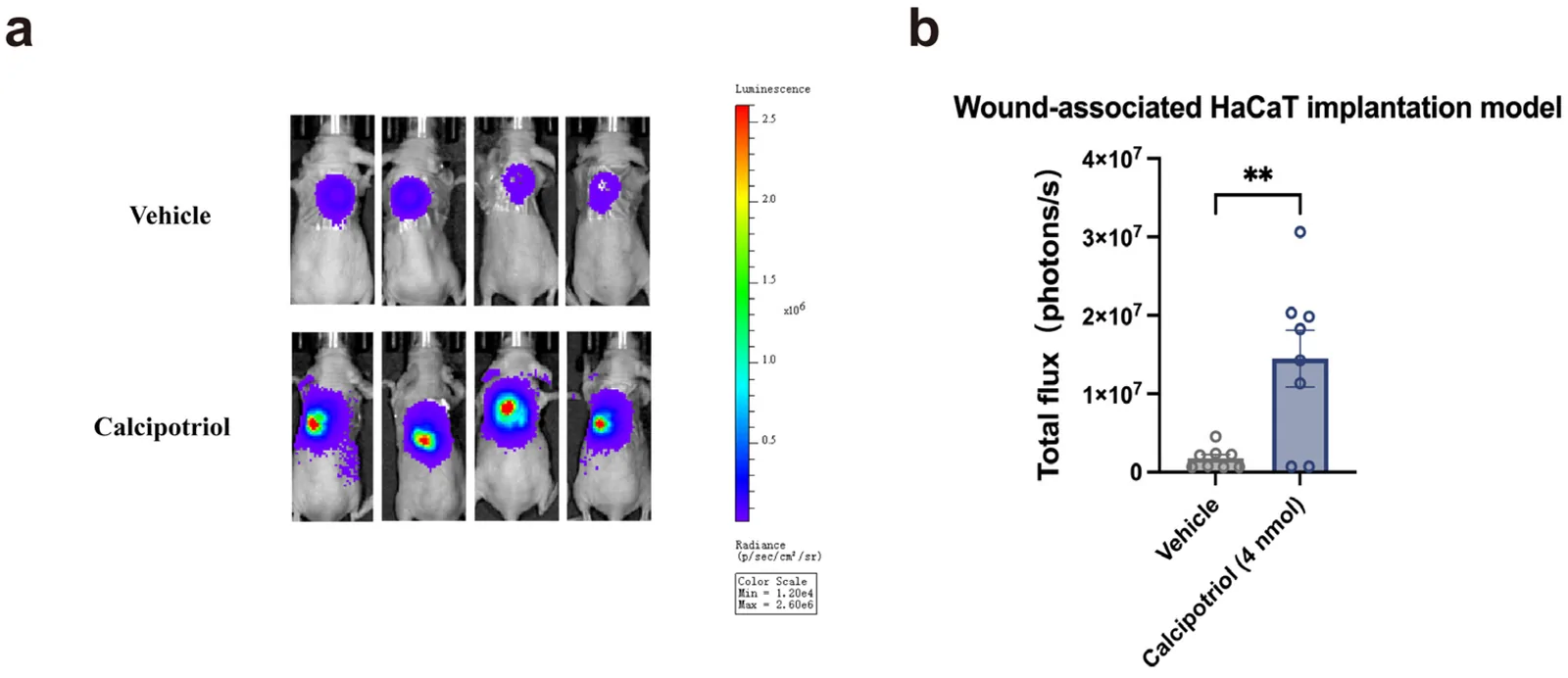
Topical calcipotriol increases bioluminescence at wound-adjacent HaCaT implantation sites. HaCaT keratinocytes stably expressing ZF10–ΔVDR and carrying the P_ZF10_–firefly luciferase reporter were suspended in 50 µL of a 1:1 mixture of PBS and Matrigel and implanted subcutaneously adjacent to 6 mm dorsal cutaneous wounds in BALB/c-nude mice (2 × 10^6^ cells per mouse). Following implantation, calcipotriol (4 nmol in 20 µL per application) or the corresponding vehicle was applied topically to the wound region twice daily for two consecutive days. At 48 h after treatment initiation, mice received D-luciferin intraperitoneally. (**a**) Representative bioluminescence images of vehicle- and calcipotriol-treated mice displayed using the same radiance scale. (**b**) Bioluminescence was quantified within a fixed region of interest as total flux (photons/s). Data are presented as mean ± SEM (*n* = 8 mice per group; each point represents one mouse). Statistical significance was determined using an unpaired two-sided *t*-test; ** *p* < 0.01.

All in vitro data are presented as the mean ± SD of three independent experiments unless otherwise specified. For the animal experiments, mice were randomly assigned to each group with comparable body weight and age. The results are presented as mean ± SEM. Unpaired *t* test, one-way ANOVA, or two-way ANOVA was used to evaluate differences among groups as indicated in the figure legends (Unpaired *t* test: Figures 4b,e and 5b; one-way ANOVA: Figures 1b–d, 2b, 3a–f and 4d, Supplementary Figures S1b–n, S3, S5a and S6a,b; two-way ANOVA: Supplementary Figure S5c). Post hoc tests are specified in the corresponding figure legends (Figures 1b–d, 2b, 3a–f and 4d, Supplementary Figures S1b–n, S3, S5a,c and S6a,b). Statistical significance was defined as *p* < 0.05. All statistical analyses were performed with GraphPad Prism version 10. Group sizes were selected based on feasibility and prior experience.

## 3. Results

### 3.1. Design and Functional Evaluation of a Calcipotriol-Inducible ZF10–ΔVDR Switch

To generate a compact calcipotriol-responsive transcriptional switch, we fused the human-derived orthogonal zinc finger array ZF10 to the hinge and ligand-binding domain of VDR, generating ZF10–ΔVDR (Figure 1a). This design preserves the ligand-responsive sensing region of VDR while removing the native VDR DNA-binding domain and N-terminal activation region, thereby reducing potential crosstalk with host genomic VDR response elements and limiting ligand-independent activation. ZF10 was selected because it recognizes a synthetic DNA sequence with no predicted high-affinity genomic match and can drive orthogonal promoter control through the P_ZF10_ promoter [7].

In HEK293T cells co-transfected with the ZF10–ΔVDR construct and a P_ZF10_-driven secreted alkaline phosphatase (SEAP) reporter, calcipotriol treatment produced concentration-dependent reporter activation (Figure 1b). To provide a functional reference within the same synthetic promoter-targeting framework, we compared ZF10–ΔVDR with the previously reported grazoprevir-responsive ZF10–NS3–p65 switch, which also operates through the ZF10/P_ZF10_ interface [7]. Using the same P_ZF10_–luciferase reporter, ZF10–NS3–p65 showed 5.5- to 15.3-fold induction across the tested grazoprevir concentrations, whereas ZF10–ΔVDR showed 11.0- to 19.3-fold induction across the tested calcipotriol concentrations (Figure 1c,d). Promoter specificity was further supported by a non-cognate P_ZF3_-SEAP reporter, which showed no appreciable calcipotriol-dependent activation by ZF10–ΔVDR (Supplementary Figure S1a,b). Together, these results indicate that direct fusion of the orthogonal ZF10 module with the hinge–ligand-binding segment of VDR yields a functional calcipotriol-inducible gene switch.

### 3.2. ZF10–ΔVDR Shows Limited Activation by Tested Vitamin D Species at Physiologically Relevant Reference Concentrations

Because endogenous vitamin D species are present in circulation, we next examined whether they could unintentionally activate the ZF10–ΔVDR system. Vitamin D3 (cholecalciferol), the precursor of downstream vitamin D metabolites, did not produce appreciable activation of ZF10–ΔVDR across 0.1–300 nM (Figure 2b). We next examined calcifediol (25-hydroxyvitamin D3), a major circulating vitamin D metabolite [30]. Across 0.1–100 nM, calcifediol did not produce appreciable activation of ZF10–ΔVDR, and only substantially higher concentrations approached measurable activation (Figure 2a). These experiments assessed reporter responses following direct compound exposure.

Because calcitriol (1,25-dihydroxyvitamin D3) is the principal endogenous ligand of VDR, we next characterized its effect on ZF10–ΔVDR. At its typical circulating concentration of approximately 100 pM [31], calcitriol produced limited activation, consistent with this concentration being well below the measured EC50 of 6.51 nM (Figure 2a; Supplementary Table S5).

Together, these direct exposure experiments showed limited ZF10–ΔVDR activation at the tested physiological reference concentrations, whereas low-nanomolar calcipotriol activated the switch.

### 3.3. ZF10–ΔVDR Functions Across Multiple Mammalian Cell Types

To evaluate the performance of ZF10–ΔVDR across different cell types, we generated stable reporter cell lines in which both the ZF10–ΔVDR construct and the P_ZF10_–luciferase reporter were stably integrated. In HaCaT keratinocytes (Figure 3a,d), HMEC-1 human dermal microvascular endothelial cells (Figure 3b,e), and BAT-SVF preadipocytes (Figure 3c,f), calcipotriol (1–3 nM) induced luciferase expression, whereas calcitriol (100 pM) produced limited activation. Calcipotriol-responsive reporter activity was also observed in murine MIN6 cells and in a human ADSC preparation (Supplementary Figure S2a,b). Notably, skin-associated cell types such as keratinocytes exhibited strong induction by calcipotriol, consistent with its established topical use.

In reporter-only HaCaT, HMEC-1, and hTERT A41h-BAT-SVF cells lacking ZF10–ΔVDR, calcipotriol did not activate P_ZF10_-driven luciferase expression but induced endogenous VDR-responsive transcription, including *CYP24A1* and cell-type-dependent *CAMP* responses (Supplementary Figure S1c–n).

Collectively, these findings show that ZF10–ΔVDR can be activated by calcipotriol across multiple human and murine cellular contexts.

### 3.4. Systemic and Topical Regulation of ZF10–ΔVDR in Implanted-Cell Model

We next assessed the acute in vivo functionality of ZF10–ΔVDR using microencapsulated HEK293T cells stably carrying the P_ZF10_–SEAP reporter and transiently expressing ZF10–ΔVDR. The cells were encapsulated in alginate–poly-L-lysine–alginate microcapsules, a semipermeable format commonly used for implantable engineered-cell systems because it retains transplanted cells while allowing diffusion of nutrients, small-molecule inducers, and secreted reporter proteins [28,29,32,33]. Encapsulated cells retained calcipotriol-responsive SEAP production in vitro under the tested short-term conditions (Supplementary Figure S3). Systemic administration of calcitriol at supraphysiological doses elevated serum SEAP signal (Figure 4a,b), indicating that the system retains responsiveness to its physiological agonist in vivo.

To explore topical induction by calcipotriol, microcapsules were implanted subcutaneously and the implantation site was treated topically with calcipotriol (Figure 4c). In the ethanol-based formulation, measurable reporter induction was observed at 4 nmol per application, whereas 0.6 nmol did not produce a detectable increase (Figure 4d). The commercial calcipotriol ointment group also showed increased reporter output. Importantly, oral supplementation with vitamin D3, the dietary precursor metabolite, did not elicit detectable activation in the same model (Figure 4e). These results indicate that ZF10–ΔVDR can be systemically activated by pharmacological calcitriol, and induced by topically applied calcipotriol at the implantation site, whereas oral vitamin D3 did not measurably activate the system under the tested two-day regimen.

### 3.5. Topical Calcipotriol Increases Reporter Activity at Wound-Adjacent HaCaT Implantation Sites

To extend the in vivo evaluation to a skin-associated engineered-cell model, HaCaT keratinocytes stably expressing ZF10–ΔVDR and carrying the P_ZF10_–luciferase reporter were implanted subcutaneously adjacent to dorsal cutaneous wounds in BALB/c-nude mice. Calcipotriol was applied topically to the wound region at 4 nmol per application, twice daily for two consecutive days. At 48 h, bioluminescence at the wound-adjacent implantation site was higher in calcipotriol-treated mice than in vehicle-treated mice (Figure 5a,b; Supplementary Figure S4). These results extend the in vivo validation of ZF10–ΔVDR to an implanted keratinocyte model and support topical calcipotriol-responsive reporter induction at a cutaneous wound site.

### 3.6. Reversible Switching and Proof-of-Concept Non-Reporter Output

**Figure 6:**
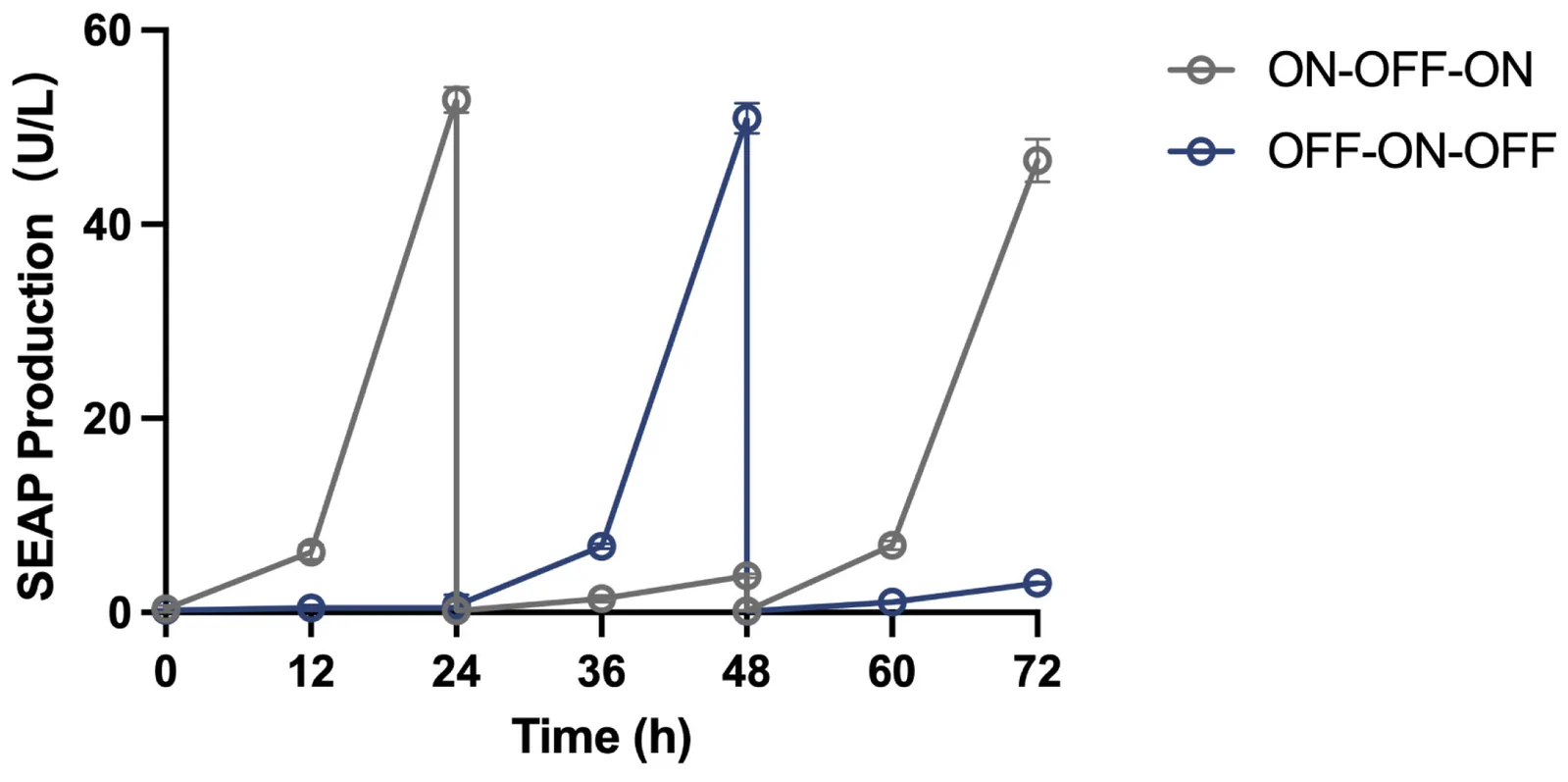
Reversible cycling behavior of the ZF10–ΔVDR reporter system. Stable HEK293T cells harboring the P_ZF10_–SEAP reporter and ZF10–ΔVDR were cultured under reciprocal ON–OFF–ON and OFF–ON–OFF schedules. Each interval lasted 24 h. In ON intervals, cells were exposed to calcipotriol for the first 6 h of the interval; in OFF intervals, cells were maintained in ligand-free medium throughout the interval. Culture supernatant samples were collected every 12 h for SEAP analysis. At the end of each 24 h interval, the preceding medium was completely removed, fresh medium corresponding to the next treatment condition was added, and cell density was readjusted. SEAP measured after each transition therefore reflected reporter secreted during the subsequent treatment interval. Data are presented as mean ± SD (*n* = 3 independent experiments). Statistical comparisons of SEAP output at 24, 48, and 72 h are provided in Supplementary Figure S6.

Because reversibility is important for regulated gene expression, we next examined whether ZF10–ΔVDR could be repeatedly cycled between ON and OFF states. Under reciprocal ON–OFF–ON and OFF–ON–OFF calcipotriol schedules, ligand withdrawal reduced reporter output during the subsequent interval, whereas re-exposure restored reporter production (Figure 6). These data support reversible ZF10–ΔVDR-mediated control under the tested cycling conditions.

To extend validation beyond reporter output, we placed hepatocyte growth factor (HGF) under ZF10–ΔVDR control and quantified secreted protein in HEK293T cells. Calcipotriol increased HGF secretion by approximately 7.2-fold at 1 nM and 15.7-fold at 3 nM relative to the untreated control (Figure S5a). In addition, in a transwell co-culture assay in which HaCaT human keratinocytes were seeded in the upper chamber and engineered HEK293T producer cells were cultured in the lower chamber, the calcipotriol-treated ZF10–ΔVDR-HGF condition was associated with increased HaCaT migration, as reflected by stronger crystal-violet staining (Figure S5b,c). These findings extend the ZF10–ΔVDR system beyond reporter expression and provide initial evidence that this switch can be coupled to a secreted protein output in vitro.

Together, these data support ZF10–ΔVDR as a compact calcipotriol-inducible gene switch that combines low basal activity, broad functionality across mammalian cell types, reversible in vitro regulation, inducible secreted protein output, and topical activation in two implanted-cell mouse models.

## 4. Discussion

Here, we report ZF10–ΔVDR as a compact calcipotriol-responsive transcriptional switch that couples ligand sensing by a truncated human VDR domain to transcriptional activation from the synthetic P_ZF10_ promoter. GAL4–VDR constructs, chemically regulated zinc-finger–steroid receptor fusions, and humanized pharmacological gene control systems have previously established the broader feasibility of combining heterologous DNA-binding modules with nuclear receptor regulatory domains [7,19,23,34]. Building on this design principle, ZF10–ΔVDR integrates the previously characterized orthogonal ZF10/P_ZF10_ promoter-targeting module [7] with the VDR hinge–ligand-binding region and uses calcipotriol as a clinically established topical VDR agonist for external control of transgene expression. The use of the human-derived ZF10 array avoids reliance on yeast-derived GAL4 DNA-binding domains and provides a compact human-derived promoter recognition module.

The ZF10/P_ZF10_ module was adopted from the synZiFTR platform, in which the ZF10 recognition sequence was designed to minimize predicted matches in the human genome and ZF10-based regulators exhibited limited perturbation of the native human transcriptome. Thus, ZF10 provides a previously characterized orthogonal DNA recognition interface for use in human cells. The present study did not independently assess genome-wide occupancy or transcriptome-wide effects of the ZF10–ΔVDR fusion; therefore, genome-wide orthogonality of the complete fusion protein was not established here.

A key challenge in using nuclear receptor domains as switch components is that their natural ligands and related metabolites are already present in vivo. For VDR-based designs, this issue is particularly important because vitamin D3, calcitriol, calcifediol, and related metabolites are part of normal vitamin D physiology [30]. The limited response of ZF10–ΔVDR at the tested physiological reference concentrations is therefore an important feature of the system under the conditions examined. These findings support low background under the tested direct exposure conditions. However, conversion of vitamin D3 and calcifediol to calcitriol was not assessed in HEK293T cells, and circulating vitamin D metabolites were not measured during the short oral vitamin D3 experiment. The contribution of metabolic conversion and tissue-specific vitamin D exposure therefore remains to be determined.

The use of calcipotriol provides a practical route for external regulation. As a topical vitamin D analog and VDR agonist with established clinical use [27], calcipotriol is attractive for engineered-cell systems located at accessible sites or for settings in which non-invasive topical dosing is preferred. In the microencapsulated-cell model, topical calcipotriol increased serum SEAP activity from subcutaneous implants, providing a systemic readout of induced reporter output. In the wound-associated HaCaT implantation model, topical calcipotriol increased bioluminescence at the treated wound and implantation site. These complementary models support topical induction of ZF10–ΔVDR-controlled transgene expression in both encapsulated HEK293T cells and skin-associated engineered keratinocytes. These experiments demonstrate induction following topical administration but do not establish spatially restricted calcipotriol exposure. Additional switch-specific implantation controls would further refine the mechanistic interpretation of the in vivo response.

The present wound-associated model demonstrates reporter induction over a 48 h treatment period but was not designed as a local or systemic safety study. The safety of repeated 4 nmol calcipotriol dosing particularly on wounded skin, remains to be established. Longer-term applications will require evaluation of local tolerability, wound healing, systemic exposure, and calcium homeostasis.

Rather than relying on native VDR response elements or non-human promoter-targeting modules, ZF10–ΔVDR redirects VDR-derived ligand sensing to the synthetic P_ZF10_ promoter through a human-derived ZF10 DNA-binding array. This design combines promoter-level orthogonality with a compact human-domain-based architecture. By replacing the endogenous VDR DNA-binding domain with the ZF10 DNA-binding array, the engineered transactivator is designed to avoid direct binding to native VDR response elements and instead activate P_ZF10_, a synthetic promoter containing ZF10-binding motifs linked to a minimal promoter. Because the ZF10-binding sequence was selected as a synthetic target with minimal predicted overlap with the human genome, the P_ZF10_ promoter is expected to be less susceptible to activation by endogenous VDR/RXR complexes or host transcriptional programs than native vitamin-D-responsive promoters. This promoter-level separation distinguishes ZF10–ΔVDR-mediated transgene regulation from endogenous VDR signaling. However, calcipotriol itself can still activate endogenous VDR signaling in responsive cells. Consistent with this separation, calcipotriol induced endogenous VDR-responsive genes in reporter-only cells but did not activate P_ZF10_-driven luciferase in the absence of ZF10–ΔVDR. Ligand-dependent activity of the retained VDR-derived region is expected to remain coupled to endogenous nuclear receptor cofactors, including RXR and transcriptional co-regulators. Thus, the ZF10–P_ZF10_ module provides promoter-level orthogonality, whereas ligand-level orthogonality is limited by the use of a VDR agonist. Future applications will therefore require attention to dose, exposure route, tissue context, and endogenous VDR activity. The activity observed in murine cell backgrounds demonstrates functional compatibility but does not independently establish orthogonality relative to the mouse genome.

The use of human-derived regulatory domains reduces reliance on bacterial, yeast, or viral transcription factor components. ZF10–ΔVDR combines a human-derived zinc-finger DNA-binding module with a human VDR-derived ligand-sensing module, providing a human-domain-based transcriptional architecture. At the same time, ZF10–ΔVDR remains mechanistically distinct from receptor-signaling-based switches because ligand sensing is coupled directly to a synthetic promoter through an engineered transcription factor rather than through endogenous signaling cascades. This direct transcriptional architecture may be useful when promoter specificity, compactness, and reduced dependence on endogenous signaling pathways are desired. The immunological properties of the fusion protein, including potential effects of the fusion junction and expression context, remain to be evaluated.

The present study primarily establishes ZF10–ΔVDR as a gene control module, while therapeutic evaluation remains a direction for future work. The reversible reporter cycling and HGF secretion experiments indicate that the switch can regulate both reporter and secreted protein outputs in vitro. However, the HGF experiment was intended as a proof-of-concept payload demonstration rather than a therapeutic efficacy study. Therapeutic applications, including dermatologic or wound repair settings, will require disease-relevant payloads, local tissue readouts, and efficacy models. Overall, ZF10–ΔVDR expands the mammalian synthetic biology toolkit as a compact calcipotriol-inducible transcriptional switch with low basal activity, limited activation by the tested endogenous vitamin D species at physiologically relevant reference concentrations, activity across multiple cellular contexts, reversible in vitro regulation, and compatibility with topical induction in implanted-cell mouse models.

## Data Availability

The data that support the findings of this study are available on reasonable request to the corresponding author.

## Supplementary Materials

The following supporting information can be downloaded at: https://www.mdpi.com/article/10.3390/genes17091072/s1. Figure S1. Promoter specificity and separation of synthetic P_ZF10_ reporter activation from endogenous VDR-responsive transcription; Figure S2. Supporting evaluation of ZF10–ΔVDR activity in additional cell backgrounds; Figure S3. Calcipotriol-responsive SEAP production after microencapsulation; Figure S4. Bioluminescence images from all animals in the wound-associated HaCaT implantation experiment; Figure S5. Calcipotriol-regulated HGF-producing cells are associated with increased HaCaT migration; Figure S6. Statistical analysis of reporter output during ON/OFF treatment cycles; Table S1. Plasmids used or constructed in this study; Table S2. DNA sequences of ZF–ΔVDR constructs and reporter cassettes; Table S3. qPCR primer sequences; Table S4. Plasmids used in each figure; Table S5. Raw concentration–response data and variable-slope curve-fitting parameters for ZF10–ΔVDR responses to calcipotriol, calcitriol, calcifediol, and vitamin D3.
