# Supplementary material for: A Calcipotriol-Responsive ZF10–ΔVDR Switch for Mammalian Transgene Regulation with Topical Induction In Vivo
Source: Genes (Basel). 2026 Sep 4;17(9):1072. doi: 10.3390/genes17091072 (PMC13606321; doi:10.3390/genes17091072)
Supplement: Supplementary file 1 [file genes-17-01072-s001.zip › genes-4474918-supplementary Figure S1-S6, Table S1-S4.pdf]

genes

MDPI

Article

A Calcipotriol-Responsive ZF10–ΔVDR Switch for Mammalian Transgene Regulation with Topical Induction In Vivo

Hong Su [1,2], Shiting Li [2,3], Guanyang Chen [1,2] and Peng Bai [2,*]

* **Correspondence:** baipeng@mail.sysu.edu.cn

Supplementary Information

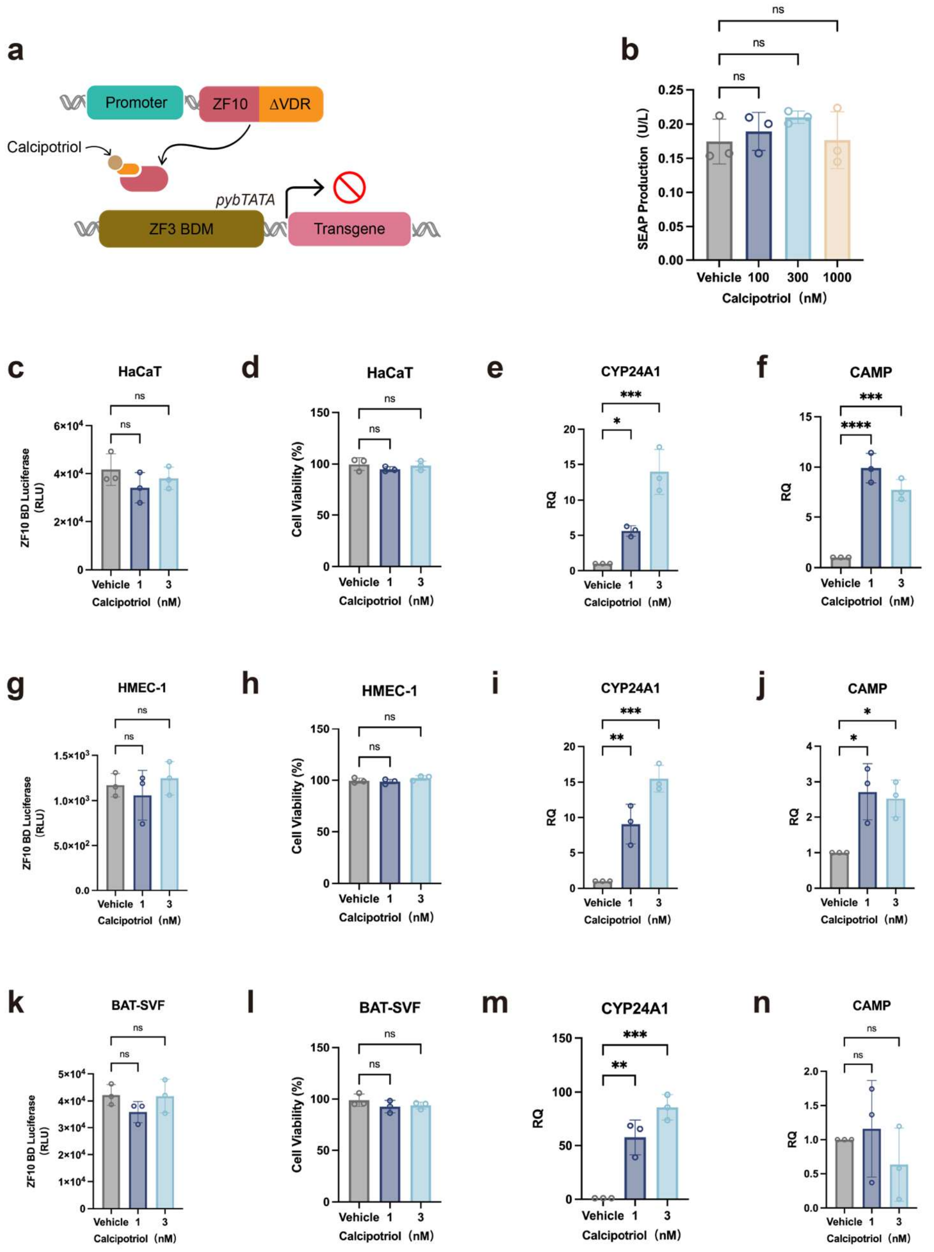

Supplementary Figure S1. Promoter specificity and separation of synthetic P_{ZF10} reporter activation from endogenous VDR-responsive transcription. (a) Schematic of the non-cognate P_{ZF3} reporter configuration. P_{ZF3} contains ZF3-binding motifs rather than the ZF10-binding motifs present in P_{ZF10}, while retaining the same minimal promoter architecture. (b) HEK293T cells expressing ZF10–ΔVDR together with the non-cognate P_{ZF3}–SEAP reporter were treated with vehicle or calcipotriol at 100, 300, or 1000 nM for 24 h, and SEAP activity was quantified. (c–f) HaCaT cells stably carrying the P_{ZF10}–luciferase reporter but lacking ZF10–ΔVDR were treated with vehicle or calcipotriol at 1 or 3 nM. (c) P_{ZF10}-driven luciferase activity. (d) Relative CCK-8 signal. (e,f) Relative expression of the endogenous VDR-responsive genes *CYP24A1* and *CAMP*, respectively, measured by RT-qPCR. (g–j) Corresponding analyses in HMEC-1 cells: P_{ZF10}-driven luciferase activity (g), relative CCK-8 signal (h), *CYP24A1* expression (i), and *CAMP* expression (j). (k–n) Corresponding analyses in hTERT A41h-BAT-SVF cells: P_{ZF10}-driven luciferase activity (k), relative CCK-8 signal (l), *CYP24A1* expression (m), and *CAMP* expression (n). Data are presented as mean ± SD (n = 3 independent experiments). Statistical significance was determined using one-way ANOVA followed by Dunnett's multiple comparisons test; ns, not significant; *$P < 0.05$; **$P < 0.01$; ***$P < 0.001$; ****$P < 0.0001$.

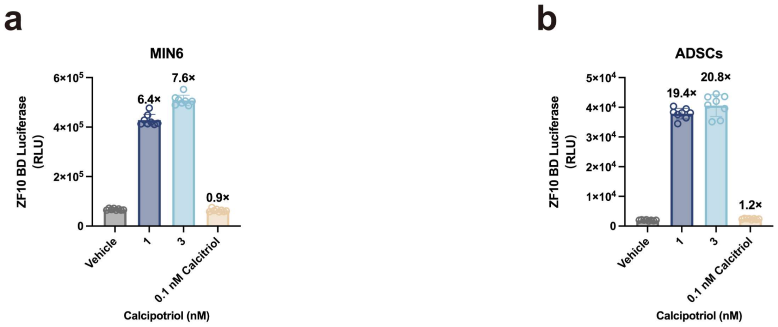

Supplementary Figure S2. Supporting evaluation of ZF10–ΔVDR activity in additional cell backgrounds. Established polyclonal MIN6 cells (a) and human ADSCs (b) carrying ZF10–ΔVDR and the P_{ZF10}–luciferase reporter were treated for 24 h with vehicle, calcipotriol at 1 or 3 nM, or calcitriol at 0.1 nM. Fold induction relative to the corresponding vehicle condition is indicated above the bars. Each panel represents one experiment using one established polyclonal population, with eight technical wells per condition. Data are shown as mean ± SD of the technical wells for descriptive purposes. The ADSC dataset was obtained using one commercial preparation (OriCell, HUXMD-01001).

https://doi.org/10.3390/genes17091072

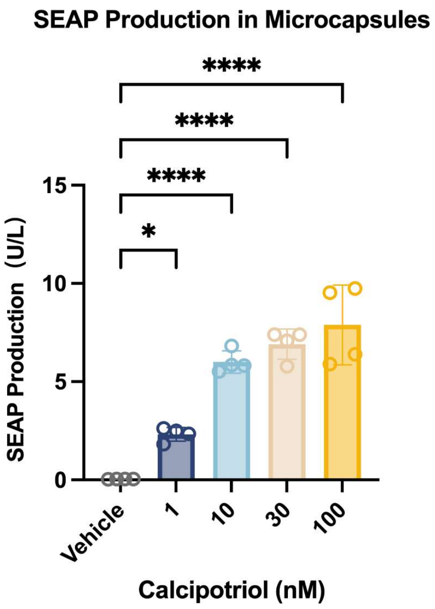

Supplementary Figure S3. Calcipotriol-responsive SEAP production after microencapsulation. HEK293T cells carrying the P_{ZF10}–SEAP reporter and expressing ZF10–ΔVDR were encapsulated in alginate–poly-L-lysine–alginate microcapsules and treated with vehicle or calcipotriol at 1, 10, 30, or 100 nM. SEAP activity in the culture supernatant was measured after 24 h. Data are presented as mean ± SD (n = 4 independent experiments). Statistical significance was determined by one-way ANOVA followed by Dunnett's multiple comparisons test; *$P < 0.05$; ****$P < 0.0001$.

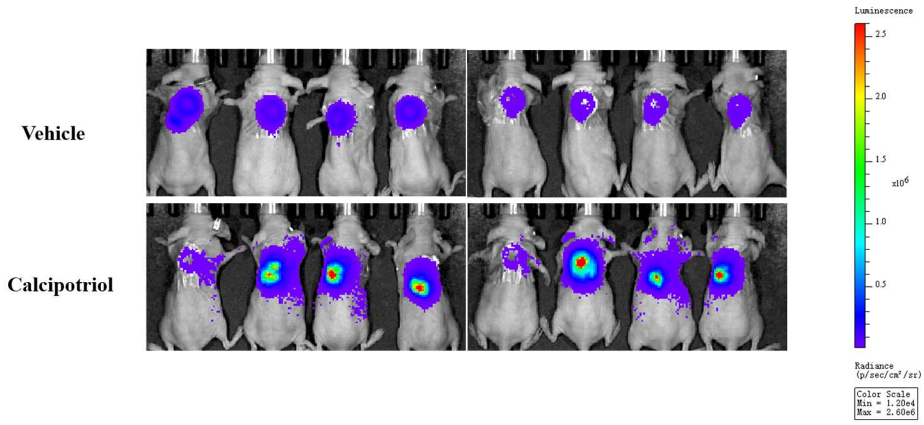

Supplementary Figure S4. Bioluminescence images from all animals in the wound-associated HaCaT implantation experiment. HaCaT cells stably expressing ZF10–ΔVDR and carrying the P_{ZF10}–firefly luciferase reporter were implanted subcutaneously adjacent to dorsal cutaneous wounds in BALB/c-nude mice. Mice received topical vehicle or calcipotriol (4 nmol per application) twice daily for two consecutive days. Bioluminescence imaging was performed 48 h after treatment initiation following intraperitoneal administration of D-luciferin. Images from all animals in the vehicle and calcipotriol groups are shown using the same radiance scale (n = 8 mice per group).

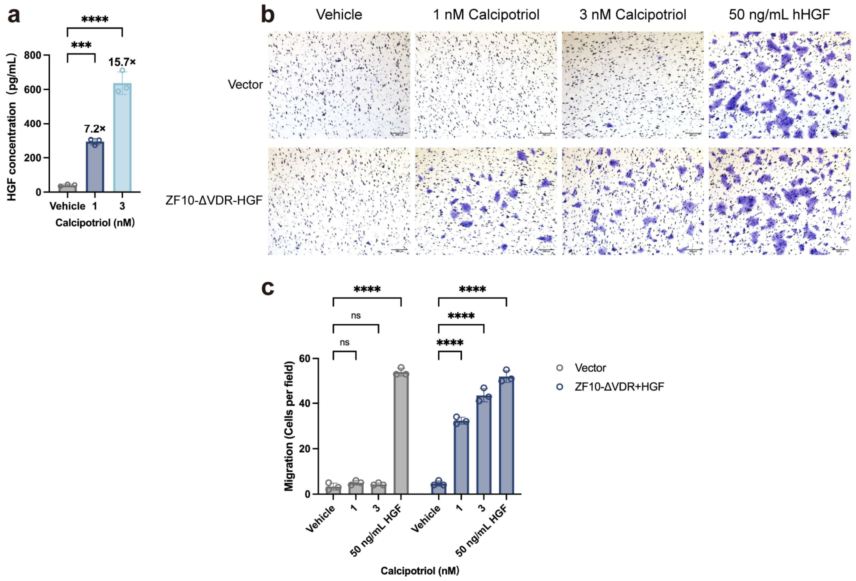

Supplementary Figure S5. Calcipotriol-regulated HGF-producing cells are associated with increased HaCaT migration. (a) Secreted HGF was quantified by ELISA in HEK293T cells engineered with ZF10–ΔVDR and a ZF10-responsive HGF output cassette after treatment with calcipotriol (1 or 3 nM) or vehicle for 24 h. Fold induction relative to the untreated control is indicated above the bars. Data are presented as mean ± SD (n = 3 independent experiments). Statistical significance was determined by one-way ANOVA followed by Dunnett's multiple comparisons test; ***P < 0.001; ****P < 0.0001. (b) Representative crystal-violet-stained images from transwell co-culture assays. HaCaT keratinocytes were seeded in the upper chamber, and vector-control or ZF10–ΔVDR–HGF producer cells were cultured in the lower chamber and treated with vehicle, 1 nM calcipotriol, or 3 nM calcipotriol. Recombinant human HGF (50 ng/mL) was included as a positive control. Scale bar, 200 μm. (c) Quantification of migrated HaCaT cells per field under the indicated conditions. Data are presented as mean ± SD (n = 3 independent experiments). Statistical significance was determined by two-way ANOVA followed by Dunnett's multiple comparisons test; ns, not significant; ****P < 0.0001.

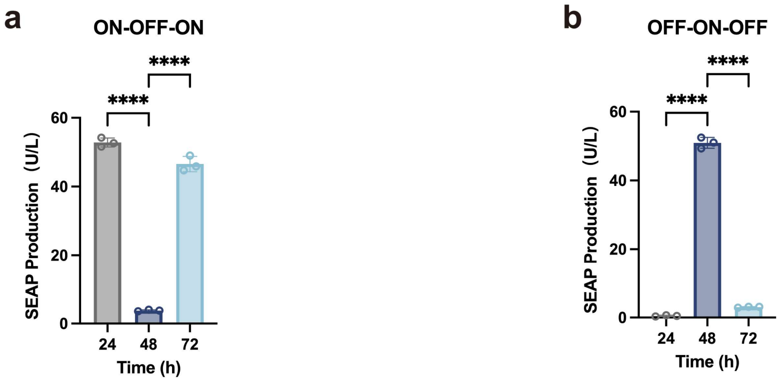

Supplementary Figure S6. Statistical analysis of reporter output during ON/OFF treatment cycles. SEAP measurements at 24, 48, and 72 h were extracted from the reciprocal ON–OFF–ON (a) and OFF–ON–OFF (b) cycling experiments shown in Figure 6. Data are presented as mean ± SD (n = 3 independent experiments). Statistical significance was determined using one-way ANOVA followed by Dunnett's multiple comparisons test; ****P < 0.0001.

Supplementary Table S1. Plasmids used or constructed in this study.

Plasmid	Description	Reference or source
pSH192	Lentiviral vector containing a P_{ZF10}-driven SEAP expression unit (LTR-P_{ZF10}-SEAP-LTR)	This study
pSH237	P_{ZF10}-driven SEAP expression vector (P_{ZF10}-SEAP-pA)	This study
pSH244	P_{ZF3}-driven SEAP expression vector (P_{ZF3}-SEAP-pA)	This study
pSH254	Lentiviral vector containing a P_{ZF10}-driven luciferase expression unit (LTR-P_{ZF10}-Luciferase-LTR)	This study
pSH257	Constitutive ZF10-NS3-p65 expression vector (P_{hCMV}-ZF10-NS3-p65-pA)	This study
pSH281	Constitutive ZF10-ΔVDR expression vector (P_{hCMV}-ZF10-ΔVDR-pA)	This study
pSH297	Lentiviral vector containing a constitutive ZF10-ΔVDR-IRES-BSD expression unit (LTR-P_{SFFV}-ZF10-ΔVDR-IRES-BSD-LTR)	This study
pGY152	P_{ZF10}-driven HGF expression unit (LTR-P_{ZF10}-HGF-LTR)	This study
pRL-SV40P	Renilla SV40 promoter (no enhancer) reporter gene for normalization of luciferase experiments	This study

Abbreviations: **BSD**, blasticidin S deaminase; **HGF**, hepatocyte growth factor; **IRES**, internal ribosome entry site; **LTR**, long terminal repeat; **NS3**, hepatitis C virus nonstructural protein 3 serine protease**; p65,** NF-κB p65 transcriptional activation domain; **pA**, polyadenylation signal; **P_{hCMV}**, human cytomegalovirus immediate early promoter; **P_{SFFV}**, spleen focus-forming virus promoter; **P_{ZF10}**, promoter with eight tandem ZF10 binding sites (for ZF10-based switches); **P_{ZF3}**, promoter with eight tandem ZF3 binding sites (for ZF3-based switches); **SEAP**, human placental secreted alkaline phosphatase; **VDR**, vitamin D receptor; **ZF10**, zinc finger 10.

Supplementary Table S2. DNA sequences of ZF–ΔVDR constructs and reporter cassettes.

Constructs	DNA sequence (5'-3')	Source
P_{hCMV}-ZF10-ΔVDR-pA	GACATTGATTATTGACTAGTTATTAA-<br>TAGTAATCAATTACGGGGTCATTAG-<br>TTCATAGCCCATATATGGAGTTCCGCGTTACATAACTT<br>ACGGTAAATGGCCCGCCTGGCTGACCGCCCAAC-<br>GACCCCCGCCCATTGACGTCAATAATGAC-<br>GTATGTTCCCATAGTAACGCCAATAGGGACTTTCCATT<br>GACGTCAATGGGTGGAGTATTTACGG-<br>TAAACTGCCCACTTGGCAGTACATCAAGTGTATCAT-<br>ATGCCAAGTACGCCCCCTATTGACGTCAATGACGGTAA<br>ATGGCCCGCCTGGCATTATGCCCAGTACATGAC-<br>CTTATGGGACTTTCCTACTTGGCAG-<br>TACATCTACGTATTAGTCATCGCTATTACCATGGTGAT<br>GCGGTTTTGGCAGTACATCAATGGGCGTGGA-<br>TAGCGGTTTGACTCACGGGGAT-<br>TTCCAAGTCTCCACCCCATTGACGTCAATGGGAGTTTG<br>TTTTGGCACCAAAATCAACGG-<br>GACTTTCCAAAATGTCGTAACAACTCCGCCCCATT-<br>GAC-<br>GCAAATGGGCGGTAGGCGTGTACGGTGGGAGGTCTAT<br>ATAAGCAGAGCTCTCTGGCTAACTAGAGAACCCAC-<br>TGCTTACTGGCTTATCGAAATTAATAC-<br>GACTCACTATAGGGAGACCCAAGCTGGCTAGCGTTTA<br>AACTTAAGCTTGCCACCATGTCTAGACCCGGA-<br>GAGCGCCCATTCCAGTGTCGGATTT-<br>GCATGCGGAACTTTTCGAGAAGACACGGCCTGGACAG<br>ACATACCCGTACTCATACAGGTGAAAAACCCTTTCAG-	This study

	TGTCGGATCTG-<br>TATGCGAAATTTCTCCGACCACAGCAGCCTGAAGAGA<br>CATCTACGTACCCACACCGGCAGCCAGAA-<br>GCCATTTCAGTGTCGGATCTGTATGCG-<br>GAACTTCTCCGTGAGACACAACCTGACCAGACATCTAC<br>GTACGCACACCGGAGAGAAGCCATTCCAATGCCGAA-<br>TATGCATGCGCAACTTCAGTGAC-<br>CACAGCAACCTGAGCAGACACCTAAAAACCCACACCG<br>GTTCCCAGAAGCCATTTCAGTGTCGGATCTGTATGCG-<br>GAACTTCTCCCAGCGCAG-<br>CAGCCTGGTGAGACATCTACGTACGCACACCGGAGAG<br>AAGCCATTCCAATGCCGAATATGCATGCG-<br>CAACTTCAGTGAGAGCGGCCACCTGAAGAGA-<br>CACCTGCGTACGCACCTGAGGGGATCCATGAAGGAGT<br>TCATTCTGACAGATGAGGAAGTGCAGAGGAAGCGG-<br>GAGATGATCCTGAAGCGGAAGGAGGAG-<br>GAGGCCTTGAAGGACAGTCTGCGGCCCAAGCTGTCTG<br>AGGAGCAGCAGCGCATCATTGCCATACTGCTGGAC-<br>GCCCACCATAAGACCTACGACCCCAC-<br>CTACTCCGACTTCTGCCAGTTCCGGCCTCCAGTTCGTG<br>TGAATGATGGTG-<br>GAGGGAGCCATCCTTCCAGGCCCAACTCCAGACACAC-<br>TCCCAGCTTCTCTGGGGACTCCTCCTCCTGCTCAG<br>ATCACTGTATCACCTCTTCAGA-<br>CATGATGGACTCGTCCAGCTTCTCCAATCTG-<br>GATCTGAG-<br>TGAAGAAGATTCAGATGACCCTTCTGTGACCCTAGAGC<br>TGTCCCAGCTCTCCATGCTGCCCCACCTGGCTGAC-	

	CTGGTCAGTTACAG-<br>CATCCAAAAGGTCATTGGCTTTGCTAAGATGATACCAG<br>GATTCAGAGACCTCACCTCTGAGGACCAGATCG-<br>TACTGCTGAAGTCAAGTGCCATT-<br>GAGGTCATCATGTTGCGCTCCAATGAGTCCTTCACCAT<br>GGACGACATGTCCTGGACCTGTGGCAACCAAGAC-<br>TACAAGTACCGCGTCAGTGACGTGAC-<br>CAAAGCCGGACACAGCCTGGAGCTGATTGAGCCCCTC<br>ATCAAGTTCCAGGTGGGACTGAAGAAGCTGAACTT-<br>GCATGAGGAGGAG-<br>CATGTCCTGCTCATGGCCATCTGCATCGTCTCCCCAGA<br>TCGTCCTGGGGTGCAGGAC-<br>GCCGCGCTGATTGAGGCCATCCAGGAC-<br>CGCCTGTCCAACACAC-<br>TGCAGACGTACATCCGCTGCCGCCACCCGCCCCCGGGC<br>AGCCACCTGCTCTATGCCAAGATGATCCAGAA-<br>GCTAGCCGACCTGCGCAGCCTCAATGAG-<br>GAGCACTCCAAGCAGTACCGCTGCCTCTCCTTCCAGCC<br>TGAGTGCAGCATGAAGCTAACGCCCCTT-<br>GTGCTCGAAGTGTTTGGCAATGA-<br>GATCTCCTGACTCGAGTCTAGAGGGCCCGTTTAAACCC<br>GCTGATCAGCCTCGACTGTGCCTTCTAGTT-<br>GCCAGCCATCTGTTGTTT-<br>GCCCCTCCCCCGTGCCTTCCTTGACCCTGGAAGGTGCC<br>ACTCCCACTGTCCTTTCCTAATAAAATGAGGAAATT-<br>GCATCGCATTGTCTGAG-<br>TAGGTGTCATTCTATTCTGGGGGGTGGGGTGGGGCAG<br>GACAGCAAGGGGGAGGATTGGGAAGACAA-<br>TAGCAGGCATGCTGGGGATGCGGTGGGCTCTATGG	

P_{ZF10}-ybTATA-SEAP	ACGATTGAGTAACACTGTATCTAGTCTAC-<br>CTACAGGGCTCGGCGTAGCCGATGTCGCGCGGA-<br>TAGCGTCAACCTCTTCGGCGTAGCCGATGTCGCGCAAT<br>CAGTTCTCCCGAGGTCGGCGTAGCCGATGTCGCG-<br>CAGAGCCGTGCTTCCAATACGGCG-<br>TAGCCGATGTCGCGCCGGGTTTCGTAACAATCGCATGA<br>GGATTCGCAACGCCTTCGGCG-<br>TAGCCGATGTCGCGCTCCCGTCTCAG-<br>TAAAGGTCGGCG-<br>TAGCCGATGTCGCGCAATCGGACTGCCTTCGTACGGCG<br>TAGCCGATGTCGCGCGTATCAGTCGCCTCGGAAC-<br>GGCGTAGCCGATGTCGCGCATTCGTAA-<br>GAGGCTCACTCTCCCTTACACGGAGTGGATAGGATCCT<br>CTAGAGGGTATATAATGGGGGCCACTAG-<br>TAGCTTCGAATCGCGAATTCGCCCAC-<br>CATGCTGCTGCTGCTGCTGCTGCTGGGCCTGAGGCTAC<br>AGCTCTCCCTGGGCATCATCCCAGTTGAGGAGGA-<br>GAACCCGGACTTCTGGAAC-<br>CGCGAGGCAGCCGAGGCCCTGGGTGCCGCCAAGAAGC<br>TGCAGCCTGCACAGACAGCCGCCAAGAAC-<br>CTCATCATCTTCCTGGGCGATGG-<br>GATGGGGGTGTCTACGGTGACAGCTGCCAGGATCCTA<br>AAAGGGCAGAAGAAGGACAAACTGGGGCCTGAGA-<br>TACCCCTGGCCATGGAC-<br>CGCTTCCCATATGTGGCTCTGTCCAAGACATACAATGT<br>AGACAAACATGTGCCAGACAGTGGAGCCACAGCCAC-<br>GGCCTACCTGTGCGGGGTCAAGGG-<br>CAACTTCCAGACCATTGGCTTGAGTGCAGCCGCCCGCT	This study

	TTAACCAGTGCAACACGACACGCGGCAAC-<br>GAGGTCATCTCCGTGATGAATCGGGCCAAGAAA-<br>GCAGGGAAGTCAGTGGGAGTGGTAACCACCACACGAG<br>TGCAGCACGCCTCGCCAGCCGGCACCTACGCCCACAC-<br>GGTGAACCGCAACTGGTACTCGGAC-<br>GCCGACGTGCCTGCCTCGGCCCGCCAGGAGGGGTGCC<br>AGGACATCGCTAC-<br>GCAGCTCATCTCCAACATGGACATTGAC-<br>GTGATCCTAGGTG-<br>GAGGCCGAAAGTACATGTTTCGCATGGGAACCCCAGA<br>CCCTGAGTACCCAGATGACTACAGCCAAGGTGGGAC-<br>CAGGCTGGACGGGAAGAATCTGGTG-<br>CAGGAATGGCTGGCGAAGCGCCAGGGTGCCCGGTATG<br>TGTGGAACCGCACTGAGCTCATGCAGGCTTCCCTG-<br>GACCCGTCTGTGACCCATCTCATGGGTCTCTTTGAGCC<br>TGGAGACATGAAATACGAGATCCACCGA-<br>GACTCCACACTGGACCCCTCCCTGATGGA-<br>GATGACAGAGGCTGCCCTGCGCCTGCTGAGCAGGAAC<br>CCCCGCGGCTTCTTCCTCTTCGTGGAGGGTGGTCG-<br>CATCGACCATGGTCATCATGAAA-<br>GCAGGGCTTACCGGGCACTGACTGAGACGATCATGTT<br>CGACGACGCCATTGAGAGGGCGGGCCAGCTCAC-<br>CAGCGAGGAGGACAC-<br>GCTGAGCCTCGTCACTGCCGACCACTCCCACGTCTTCT<br>CCTTCGGAGGC-<br>TACCCCCTGCGAGGGAGCTCCATCTTCGGGCTGGCCCC<br>TGGCAAGGCCCGGGACAGGAAGGCCTACAC-<br>GGTCCTCCTATACGGAAACGGTCCAGGC-<br>TATGTGCTCAAGGACGGCGCCCGGCCGGATGTTACCG	

	AGAGCGAGAGCGGGAGCCCCGAGTATCGGCAGCAG-<br>TCAGCAGTGCCCCTGGACGAAGA-<br>GACCCACGCAGGCGAGGACGTGGCGGTGTTCGCGCGC<br>GGCCCGCAGGCGCACCTGGTTCACGGCGTGCAGGAG-<br>CAGACCTTCATAGCGCAC-<br>GTCATGGCCTTCGCCGCCTGCCTGGAGCCCTACACCGC<br>CTGCGACCTGGCGCCCCCGCCGGCACCACCGAC-<br>GCCGCGCACCCGGGTTACTCTAGAG-<br>TCGGGGCGGCCGGCCGCTTCGAGCAGACATGA	
P_{ZF10}-ybTATA-Lucif-<br>erase	ACGATTGAGTAACACTGTATCTAGTCTAC-<br>CTACAGGGCTCGGCGTAGCCGATGTCGCGCGGA-<br>TAGCGTCAACCTCTTCGGCGTAGCCGATGTCGCGCAAT<br>CAGTTCTCCCGAGGTCGGCGTAGCCGATGTCGCG-<br>CAGAGCCGTGCTTCCAATACGGCG-<br>TAGCCGATGTCGCGCCGGGTTTCGTAACAATCGCATGA<br>GGATTCGCAACGCCTTCGGCG-<br>TAGCCGATGTCGCGCTCCCGTCTCAG-<br>TAAAGGTCGGCG-<br>TAGCCGATGTCGCGCAATCGGACTGCCTTCGTACGGCG<br>TAGCCGATGTCGCGCGTATCAGTCGCCTCGGAAC-<br>GGCGTAGCCGATGTCGCGCATTCGTAA-<br>GAGGCTCACTCTCCCTTACACGGAGTGGATAGGATCCT<br>CTAGAGGGTATATAATGGGGGCCACTAGTGCCAC-<br>CATGGAAGATGCCAAAAACATTAA-<br>GAAGGGCCCAGCGCCATTCTACCCACTCGAAGACGGG<br>ACCGCCGGCGAGCAGCTGCACAAAGCCATGAAGCGC-<br>TACGCCCTGGTGCCCGGCAC-<br>CATCGCCTTTACCGACGCACATATCGAGGTGGACATTA	This study

	CCTACGCCGAGTACTTCGAGATGAGCGTTCGGCTGG-<br>CAGAAGCTATGAAGCGC-<br>TATGGGCTGAATACAAACCATCGGATCGTGGTGTGCA<br>GCGAGAATAGCTTGCAGTTCTTCATGCCCGTGTT-<br>GGGTGCCCTGTTCATCGGTGTGGCTGTGGCCCCAGCTA<br>ACGACATCTACAACGAGCGCGAGCTGCTGAACAG-<br>CATGGGCATCAGCCAGCCCACCGTCG-<br>TATTCGTGAGCAAGAAAGGGCTGCAAAAGATCCTCAA<br>CGTGCAAAAGAAGCTACCGATCATACAAAA-<br>GATCATCATCATGGATAGCAAGACCGACTAC-<br>CAGGGCTTCCAAAGCATGTACACCTTCGTGACTTCCCA<br>TTTGCCACCCGGCTTCAACGAGTAC-<br>GACTTCGTGCCCGAGAGCTTCGACCGGGACAAAAC-<br>CATCGCCCTGATCATGAACAGTAGTGGCAGTACCGGA<br>TTGCCCAAGGGCGTAGCCCTACCGCACCGCACCGCTT-<br>GTGTCCGATTCAG-<br>TCATGCCCGCGACCCCATCTTCGGCAACCAGATCATCC<br>CCGACACCGCTATCCTCAGCGTGGTGCCATTTCAC-<br>CACGGCTTCGGCATGTTCACCAC-<br>GCTGGGCTACTTGATCTGCGGCTTTCGGGTCGTGCTCA<br>TGTACCGCTTCGAGGAGGAGCTATTCTTGCGCAGCTT-<br>GCAAGACTATAAGAT-<br>TCAATCTGCCCTGCTGGTGCCCACACTATTTAGCTTCTT<br>CGCTAAGAGCACTCTCATCGACAAGTACGACCTAA-<br>GCAACTTGCACGA-<br>GATCGCCAGCGGCGGGGCGCCGCTCAGCAAGGAGGTA<br>GGTGAGGCCGTGGCCAAACGCTTCCACCTAC-<br>CAGGCATCCGCCAGGGCTAC-<br>GGCCTGACAGAAACAACCAGCGCCATTCTGATCACCC	

	CCGAAGGGGACGACAAGCCTGGCGCAG-TAGGCAAGGTGGTGCCCTTCTTCGAGGCTAAGGTGGTGGACTTGGACACCGGTAAGACACTGGGTGTGAAC-CAGCGCGGCGAGCTGTGCGTCCGTGGCCCCATGATCATGAGCGGCTACGTTAACAACCCCGAGGCTACAAAC-GCTCTCATCGACAAGGACGGCTGGCTG-CACAGCGGCGACATCGCCTACTGGGACGAGGACGAGCACTTCTTCATCGTGGACCGGCTGAA-GAGCCTGATCAAATACAAGGGCTAC-CAGGTAGCCCCAGCCGAACTGGAGAGCATCCTGCTGCAACACCCCAACATCTTCGAC-GCCGGGGTCGCCGGCCTGCCCGACGAC-GATGCCGGCGAGCTGCCCGCCGCAGTCGTCGTGCTGGAACACGGTAAAACCATGACCGAGAAGGAGATCGTG-GACTATGTGGCCAGCCAGGTTACAAC-CGCCAAGAAGCTGCGCGGTGGTGTTGTGTTCGTGGACGAGGTGCCTAAAGGACTGACCGGCAAGTTGGAC-GCCCGCAAGATCCGCGAGAT-TCTCATTAAGGCCAAGAAGGGCGGCAAGATCGCCGTGTAA	
P_{ZF3}-ybTATA-SEAP	ACGATTGAGTAACACTGTATCTAGTCTAC-CTACAGGGCTAGACGTCGAAGTAGCCGTAGGGA-TAGCGTCAACCTCTTAGACGTCGAAGTAGCCGTAGAATCAGTTCTCCCGAGGTAGACGTCGAAGTAGCCGTAGA-GAGCCGTGCTTCCAATAAGAC-GTCGAAGTAGCCGTAGCGGGTTTCGTAACAATCGCATGAGGATTCGCAACGCCTTAGACGTCGAAGTAGCCG-TAGTCCCGTCTCAGTAAAGGTAGAC-GTCGAAGTAGCCGTAGAATCGGACTGCCTTCGTAAGA	This study

	CGTCGAAGTAGCCGTAGGTATCAGTCGCCTCGGAAA-<br>GACGTCGAAGTAGCCGTAGATTCG-<br>TAAGAGGCTCACTCTCCCTTACACGGAGTGGATAGGAT<br>CCTCTAGAGGGTATATAATGGGGGCCAAA-<br>GCTTCGAATCGCGAATTCGCCCAC-<br>CATGCTGCTGCTGCTGCTGCTGCTGGGCCTGAGGCTAC<br>AGCTCTCCCTGGGCATCATCCCAGTTGAGGAGGA-<br>GAACCCGGACTTCTGGAAC-<br>CGCGAGGCAGCCGAGGCCCTGGGTGCCGCCAAGAAGC<br>TGCAGCCTGCACAGACAGCCGCCAAGAAC-<br>CTCATCATCTTCCTGGGCGATGG-<br>GATGGGGGTGTCTACGGTGACAGCTGCCAGGATCCTA<br>AAAGGGCAGAAGAAGGACAAACTGGGGCCTGAGA-<br>TACCCCTGGCCATGGAC-<br>CGCTTCCCATATGTGGCTCTGTCCAAGACATACAATGT<br>AGACAAACATGTGCCAGACAGTGGAGCCACAGCCAC-<br>GGCCTACCTGTGCGGGGTCAAGGG-<br>CAACTTCCAGACCATTGGCTTGAGTGCAGCCGCCCGCT<br>TTAACCAGTGCAACACGACACGCGGCAAC-<br>GAGGTCATCTCCGTGATGAATCGGGCCAAGAAA-<br>GCAGGGAAGTCAGTGGGAGTGGTAACCACCACACGAG<br>TGCAGCACGCCTCGCCAGCCGGCACCTACGCCCACAC-<br>GGTGAACCGCAACTGGTACTCGGAC-<br>GCCGACGTGCCTGCCTCGGCCCGCCAGGAGGGGTGCC<br>AGGACATCGCTAC-<br>GCAGCTCATCTCCAACATGGACATTGAC-<br>GTGATCCTAGGTG-<br>GAGGCCGAAAGTACATGTTTCGCATGGGAACCCCAGA	

	CCCTGAGTACCCAGATGACTACAGCCAAGGTGGGAC-<br>CAGGCTGGACGGGAAGAATCTGGTG-<br>CAGGAATGGCTGGCGAAGCGCCAGGGTGCCCGGTATG<br>TGTGGAACCGCACTGAGCTCATGCAGGCTTCCCTG-<br>GACCCGTCTGTGACCCATCTCATGGGTCTCTTTGAGCC<br>TGGAGACATGAAATACGAGATCCACCGA-<br>GACTCCACACTGGACCCCTCCCTGATGGA-<br>GATGACAGAGGCTGCCCTGCGCCTGCTGAGCAGGAAC<br>CCCCGCGGCTTCTTCCTCTTCGTGGAGGGTGGTCG-<br>CATCGACCATGGTCATCATGAAA-<br>GCAGGGCTTACCGGGCACTGACTGAGACGATCATGTT<br>CGACGACGCCATTGAGAGGGCGGGCCAGCTCAC-<br>CAGCGAGGAGGACAC-<br>GCTGAGCCTCGTCACTGCCGACCACTCCCACGTCTTCT<br>CCTTCGGAGGC-<br>TACCCCCTGCGAGGGAGCTCCATCTTCGGGCTGGCCCC<br>TGGCAAGGCCCGGGACAGGAAGGCCTACAC-<br>GGTCCTCCTATACGGAAACGGTCCAGGC-<br>TATGTGCTCAAGGACGGCGCCCGGCCGGATGTTACCG<br>AGAGCGAGAGCGGGAGCCCCGAGTATCGGCAGCAG-<br>TCAGCAGTGCCCCTGGACGAAGA-<br>GACCCACGCAGGCGAGGACGTGGCGGTGTTCGCGCGC<br>GGCCCGCAGGCGCACCTGGTTCACGGCGTGCAGGAG-<br>CAGACCTTCATAGCGCAC-<br>GTCATGGCCTTCGCCGCCTGCCTGGAGCCCTACACCGC<br>CTGCGACCTGGCGCCCCCCGCCGGCACCACCGAC	

Supplementary Table S3. qPCR Primer Sequences.

Gene	Sequence (5'-3')
CYP24A1-F	GCTTCTCCAGAAGAATGCAGGG

CYP24A1-R	CAGACCTTGGTGTTGAGGCTCT
CAMP-F	GACACAGCAGTCACCAGAGGAT
CAMP-R	TCACAACTGATGTCAAAGGAGCC
GAPDH-F	GTCTCCTCTGACTTCAACAGCG
GAPDH-R	ACCACCCTGTTGCTGTAGCCAA

Supplementary Table S4. Plasmids used in each figure.

**Figure 1**	
b	pSH281, pSH237
c-d	pSH257, pSH254; pSH281, pSH254

**Figure 2**	
a-b	pSH297, pSH192

**Figure 3**	
a-f	pSH297, pSH254

**Figure 4**	
b,d,e	pSH297, pSH192

**Figure 5**	
	pSH297, pSH254

**Figure 6**	
	pSH297, pSH192

**Figure S1**	
a-b	pSH281, pSH244
c-n	pSH254

**Figure S2**	

a-b	pSH297, pSH254

**Figure S3**	
	pSH297, pSH192

**Figure S4**	
	pSH297, pSH254

**Figure S5**	
a-c	pSH297, pGY152

**Figure S6**	
a-b	pSH297, pSH192

https://doi.org/10.3390/genes17091072
